# Elaidic acid drives cellular senescence and inflammation via lipid raft-mediated IL-1R signaling

**DOI:** 10.1016/j.isci.2025.113305

**Published:** 2025-08-06

**Authors:** Ryota Kojima, Yusuke Hirata, Ryo Ashida, Miki Takahashi, Ryosuke Matsui, Kotaro Hama, Ayako Watanabe, Ryo Takita, Emiko Sato, Taiki Abe, Kazuaki Yokoyama, Takuya Noguchi, Atsushi Matsuzawa

**Affiliations:** 1 Laboratory of Health Chemistry, Graduate School of Pharmaceutical Sciences, Tohoku University, 6-3 Aoba, Aramaki, Aoba-ku, Sendai 980-8578, Japan; 2 Faculty of Pharmaceutical Sciences, Teikyo University, 2-11-1 Kaga, Itabashi-ku, Tokyo 173-8605, Japan; 3 Advanced Comprehensive Research Organization (ACRO), Teikyo University, 2-11-1 Kaga, Itabashi-ku, Tokyo 173-8605, Japan; 4 One-stop Sharing Facility Center for Future Drug Discoveries, Graduate School of Pharmaceutical Sciences, University of Tokyo, 7-3-1 Hongo, Bunkyo-ku, Tokyo 113-0033, Japan; 5 School of Pharmaceutical Sciences, University of Shizuoka, 52-1 Yada, Suruga-ku, Shizuoka 422-8526, Japan; 6 Division of Clinical Pharmacology and Therapeutics, Graduate School of Pharmaceutical Sciences, Tohoku University, 6-3 Aoba, Aramaki, Aoba-ku, Sendai 980-8578, Japan; 7 Department of Medical Genetics, Tohoku University School of Medicine, 1-1 Seiryo-machi, Aoba-ku, Sendai 980-8574, Japan; 8 Department of Medical Biochemistry, School of Pharmacy, Iwate Medical University, 1-1-1 Idaidori, Yahaba-cho, Shiwa-gun, Iwate Prefecture 028-3694, Japan

**Keywords:** Biochemistry, Cell biology

## Abstract

*trans-*Fatty acids (TFAs) have been associated with various inflammatory diseases, including atherosclerosis and metabolic syndrome, such as metabolic dysfunction-associated steatotic liver disease (MASLD)/metabolic dysfunction-associated steatohepatitis (MASH). However, the underlying mechanism remains unclear. Here, we show that in response to DNA damage, elaidic acid (EA), a most common TFA, amplifies interleukin-1 receptor (IL-1R) signaling, leading to the promotion of cellular senescence and senescence-associated secretory phenotype (SASP). Upon DNA damage, EA enhanced senescence-associated β-galactosidase activity and expressions of IL-1α/6/8 through the IL-1R-transforming growth factor-β-activated kinase 1 (TAK1)-nuclear factor (NF)-κB axis in a manner dependent on mammalian target of rapamycin (mTOR). Mechanistically, EA, incorporated into lipid rafts, enhances IL-1R activation and subsequent NF-κB signaling, creating a positive feedback loop. EA consumption elevated expressions of SASP factors and cellular senescence in the livers of high-fat diet mice. Our findings provide a mechanistic insight into TFA-related inflammation and disorders, including MASLD/MASH.

## Highlights

• Elaidic acid (EA) facilitates cellular senescence and inflammation upon DNA damage
• EA integrates into lipid rafts, promoting IL-1R enrichment within raft microdomains
• EA sensitizes IL-1R, thereby amplifying proinflammatory TAK1-NF-κB signaling
• Dietary EA promotes hepatic cellular senescence and inflammation in MASLD mice

## Graphical abstract

**Figure:**
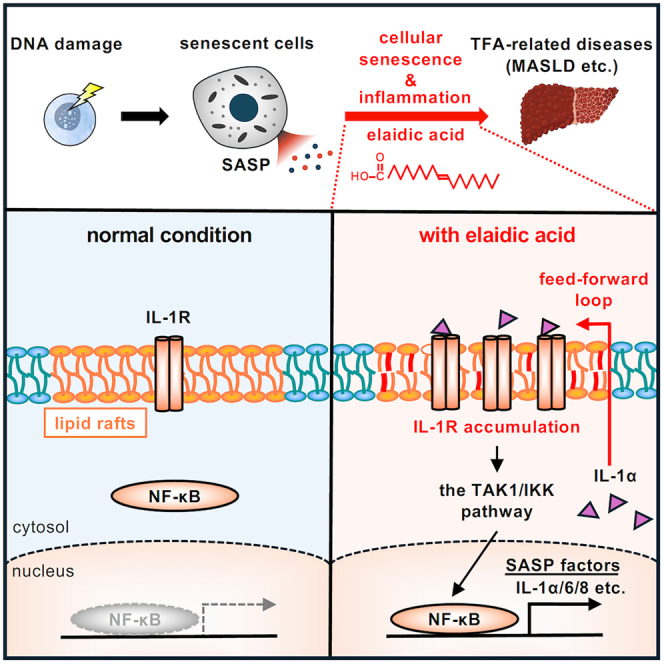

## Introduction

*trans*-Fatty acids (TFAs) are unsaturated fatty acids (UFAs) characterized by the presence of one or more trans double bonds in their hydrocarbon chains. Since fatty acid desaturases introduce double bonds only in the *cis* configuration in our body, endogenously synthesized UFAs exclusively contain *cis* double bonds, henceforth referred to as *cis*-fatty acids (CFAs).^1^^,^^2^ On the other hand, TFAs are primarily obtained through dietary intake. There are five major TFA species in foods, which are classified into two types based on the difference in their origins: industrial TFAs (iTFAs) and ruminant TFAs (rTFAs)^2^ (Figure S1A). iTFAs, including elaidic acid (EA, C18:1 t9) and linoelaidic acid (LEA, C18:2 t9 t12), are predominantly present in processed foods, including snacks and fast foods, which are formed as byproducts during industrial food processing, such as the partial hydrogenation of CFAs contained in edible oils. In contrast, rTFAs, such as *trans*-vaccenic acid (TVA, C18:1 t11), rumenic acid (RA, C18:2 c9 t11), and palmitelaidic acid (PEA, C16:1 t9), are rich in meat and dairy products derived from ruminant animals, including cows and sheep, in which CFAs are constitutively subjected to bacterial biohydrogenation.^2^ A growing body of epidemiological evidence suggests that the intake of iTFAs is associated with an increased risk of various disorders, such as cardiovascular diseases (CVDs), neurodegenerative diseases (NDs), metabolic syndrome, and systemic inflammation, as evidenced by elevated levels of inflammatory markers such as C-reactive protein (CRP) and interleukin-6 (IL-6).^3^^,^^4^^,^^5^^,^^6^ Among these, atherosclerosis, a leading cause of CVDs, has been strongly associated with the consumption of iTFAs, whose detrimental effects have been mostly attributed to their particular adverse impact on lipoprotein metabolism, elevated low-density lipoprotein cholesterol (LDL-C) levels, and reduced high-density lipoprotein cholesterol (HDL-C) levels in blood.^4^^,^^7^^,^^8^ Furthermore, we have recently revealed pro-apoptotic activities unique to iTFAs in response to a damage-associated molecular pattern (DAMP), extracellular ATP (eATP), and DNA damage.^9^^,^^10^^,^^11^^,^^12^ Upon eATP stimulation, iTFAs facilitate activation of the apoptosis signal-regulating kinase 1 (ASK1)-p38 mitogen-activated protein (MAP) kinase pathway, whereas they potentiate the generation of reactive oxygen species (ROS) via mitochondria and NADPH oxidase during DNA damage, thereby promoting apoptosis.^9^^,^^10^^,^^11^^,^^12^ Importantly, these toxic actions were specifically observed with iTFAs, including EA and LEA, but not with their geometrical isomers, namely oleic acid (OA, C18:1 c9) and linoleic acid (LA, C18:2 c9 c12), palmitic acid (PA, C16:0) as the most abundant saturated fatty acid (SFA) in our body, or rTFAs including TVA, RA, and PEA.^13^^,^^14^ Thus, while the atherogenic and pro-apoptotic effects unique to iTFAs can well explain their association with CVDs and NDs, the mechanisms underlying their contribution to other TFA-related disorders, including systemic inflammation and metabolic syndrome, remain unclear.

DNA damage, induced by various intracellular and extracellular stressors, triggers a cellular response aimed at maintaining cellular homeostasis.^15^ When DNA damage is beyond the capacity of DNA repair system, cells undergo apoptosis, while moderate damage may induce cellular senescence.^15^ Cellular senescence was once viewed only as a tumor-suppressive mechanism, but recent studies have established that senescent cells secrete a variety of inflammatory factors, a phenomenon known as the senescence-associated secretory phenotype (SASP).^16^ The nuclear factor (NF)-κB signaling pathway plays a crucial role in the induction of SASP.^16^ The activation of NF-κB leads to the transcription of a variety of pro-inflammatory cytokines and chemokines, such as IL-1, IL-6, and IL-8, collectively known as SASP factors, which contribute to a feedforward loop, amplifying cellular senescence.^16^ While SASP has beneficial effects, such as promoting tissue repair, it can also contribute to chronic inflammation and the development of age-related diseases, including CVDs, NDs, and metabolic syndrome.^16^ Intriguingly, it has been reported that the treatment of human endothelial cells with iTFAs promotes NF-κB activation and subsequent induction of pro-inflammatory cytokines,^17^ and that iTFA administration to peroxisome proliferator-activated receptor α (PPARα)-deficient mice exacerbates metabolic dysfunction-associated steatotic liver disease (MASLD)/metabolic dysfunction-associated steatohepatitis (MASH) with increased NF-κB activation and inflammation.^18^ However, the underlying molecular mechanisms are still unknown.

Herein, we report a mechanism by which EA, the most abundant iTFA, promotes inflammation through cellular senescence and SASP induced by DNA damage. EA was incorporated into lipid rafts, facilitating the activation of the IL-1 receptor (IL-1R)-NF-κB signaling, which in turn promoted the induction of SASP factors such as IL-1 and IL-8, thereby amplifying cellular senescence. This effect was not observed with OA as a *cis* isomer of EA. Furthermore, in a mouse model of MASLD/MASH, EA intake exacerbated cellular senescence and SASP in the liver. These findings provide a mechanistic insight into the mechanisms underlying TFA-related disorders associated with inflammation.

## Results

### Elaidic acid promotes DNA damage-induced cellular senescence and senescence-associated secretory phenotype

**Figure 1:**
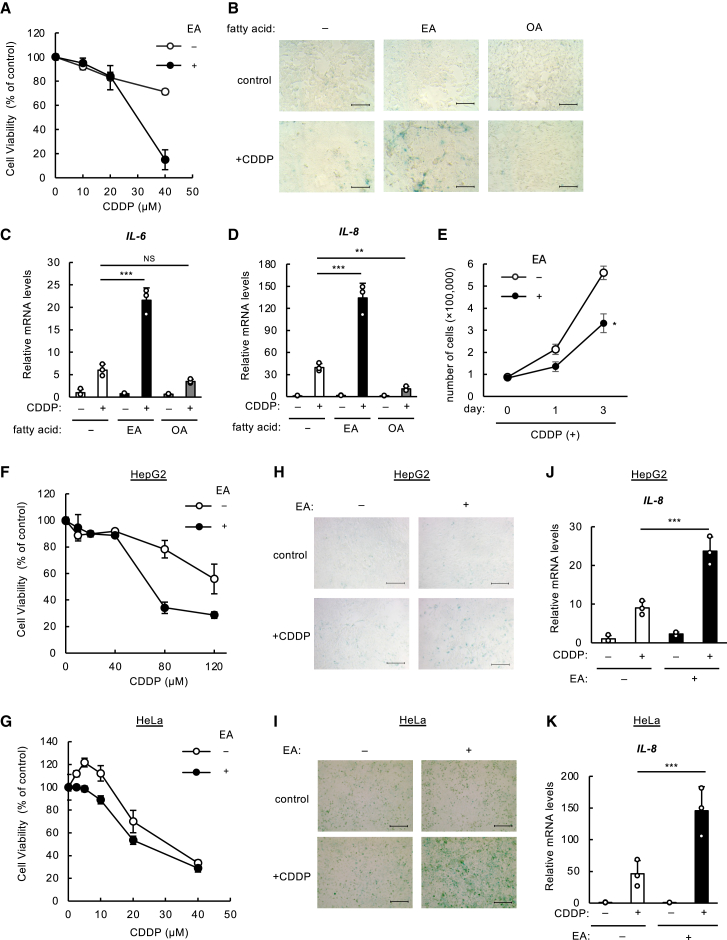
EA promotes DNA damage-induced cellular senescence and SASP (A) U2OS cells were pretreated with or without 200 μM EA for 12 h, stimulated with various concentrations of CDDP for 24 h, and assayed for cell viability. Data shown are the mean ± SD (*n* = 3). (B) U2OS cells were pretreated with 200 μM EA or OA for 12 h, and then stimulated with 2 μM CDDP. After 48 h, the medium was replaced with a new medium with the same concentration of fatty acids and cultured for additional 5 days before being subjected to SA-β-gal staining. Scale bar, 100 μm (C and D) U2OS cells were pretreated with or without 200 μM EA for 12 h, and then stimulated with 20 μM CDDP for 48 h, subjected to qRT-PCR analysis. Relative mRNA levels of *IL-6* and *IL-8* are shown as mean ± SD (*n* = 3). (E) U2OS cells were pretreated with or without 200 μM EA for 12 h, followed by treatment with 2 μM CDDP for 48 h. After CDDP treatment, the medium was replaced with fresh medium with or without EA (day 0), and cells were cultured, harvested, and counted at the indicated days. (F and G) HepG2 and HeLa cells were pretreated with or without 200 μM EA for 12 h, stimulated with various concentrations of CDDP for 24 h, and assayed for cell viability assay. Data shown are the mean ± SD (*n* = 3). (H and I) HepG2 and HeLa cells were pretreated with or without 200 μM EA for 12 h, and then stimulated with 0.25 μM and 1.25 μM CDDP, respectively. After 48 h, the medium was replaced with a new medium with the same concentration of fatty acids and cultured for 5 days and 3 days, respectively. The cells were then subjected to SA-β-gal staining. Scale bar, 100 μm (J and K) HepG2 cells and HeLa cells were pretreated with or without 200 μM EA for 12 h, and then stimulated with 5 μM or 10 μM CDDP for 48 h respectively, subjected to qRT-PCR analysis. Relative mRNA levels of *IL-8* are shown as mean ± SD (*n* = 3). See also Figure S1.

We have previously demonstrated that EA plays a pro-apoptotic role upon high-dose treatment with DNA-damaging agents such as cisplatin (CDDP) in several cell lines, mainly in U2OS, human osteosarcoma cells.^12^ To investigate the effect of EA on cellular senescence, which is generally triggered by a sub-lethal degree of DNA damage,^15^ we first checked the dose response of U2OS to CDDP with or without EA. While we consistently observed a clear decrease in cell survival upon EA pretreatment along with high-dose CDDP treatment (40 μM), no significant change was observed when treated with a sub-lethal dose of CDDP, at concentrations below 20 μM (Figure 1A). We hereafter treated U2OS cells with a sub-lethal dose of CDDP, not more than 20 μM, to induce senescence. U2OS cells were pretreated with EA or OA, as the *cis*-isomer of EA, followed by treatment with CDDP, and subjected to senescence associated-β galactosidase (SA-β-gal) staining, a representative method for detecting senescent cells.^19^ As shown in Figures 1B and S5A, pretreatment with EA, but not OA, robustly increased SA-β-gal-positive cells in response to CDDP, indicative of the promotion of senescence. Senescent cells secrete pro-inflammatory cytokines and chemokines, such as IL-6 and IL-8, respectively, and thereby induce inflammation, which is well known as SASP.^15^ As expected, mRNA levels of SASP factors, IL-6 and IL-8, were also elevated in the presence of EA plus CDDP (Figures 1C and 1D). Delayed proliferation and formation of senescence-associated heterochromatin foci (SAHF) are hallmark features of senescent cells.^20^ As shown in Figure 1E and S1B, pretreatment with EA suppressed cell proliferation, while promoting SAHF formation under sub-lethal CDDP treatment. Consistently, we observed that EA treatment elevated the expression of p21, a key marker of cell-cycle arrest (Figure S1C).^20^ These results collectively suggest that EA plays a pro-senescence role in response to DNA damage upon CDDP treatment.

There are five major TFA species in foods, EA and LEA as typical iTFAs and TVA, RA, and PEA as typical rTFAs (Figure S1A).^2^ To test which of the five major TFAs works as a promoter of senescence, we pretreated U2OS cells with these TFAs and examined their effect on SASP induction. We found that EA was the only TFA that could potently enhance IL-8 induction in response to CDDP (Figure S1D), suggesting that EA has a unique role as a senescence-promoting fatty acid among the five major TFAs. EA facilitated SASP in response to other DNA damage inducers than CDDP, including 1-Methyl-3-nitro-1-nitrosoguanidine (MNNG) and H_2_O_2_ (Figure S1E). Furthermore, in other cell types than U2OS cells, including HepG2 (human liver cancer cells) and HeLa (human cervical cancer cells), upon sub-lethal doses of CDDP, at below 5 μM and 10 μM, respectively (Figures 1F and 1G), EA pretreatment substantially elevated the number of SA-β-gal-positive cells (Figures 1H and 1I; S5B and S5C) and IL-8 mRNA levels (Figures 1J and 1K). These data collectively suggest that pro-senescence activity of EA is commonly observed across various cell lines upon multiple types of DNA damage.

### The mTOR complex 1-NF-κB axis involves pro-senescence activity of elaidic acid

**Figure 2:**
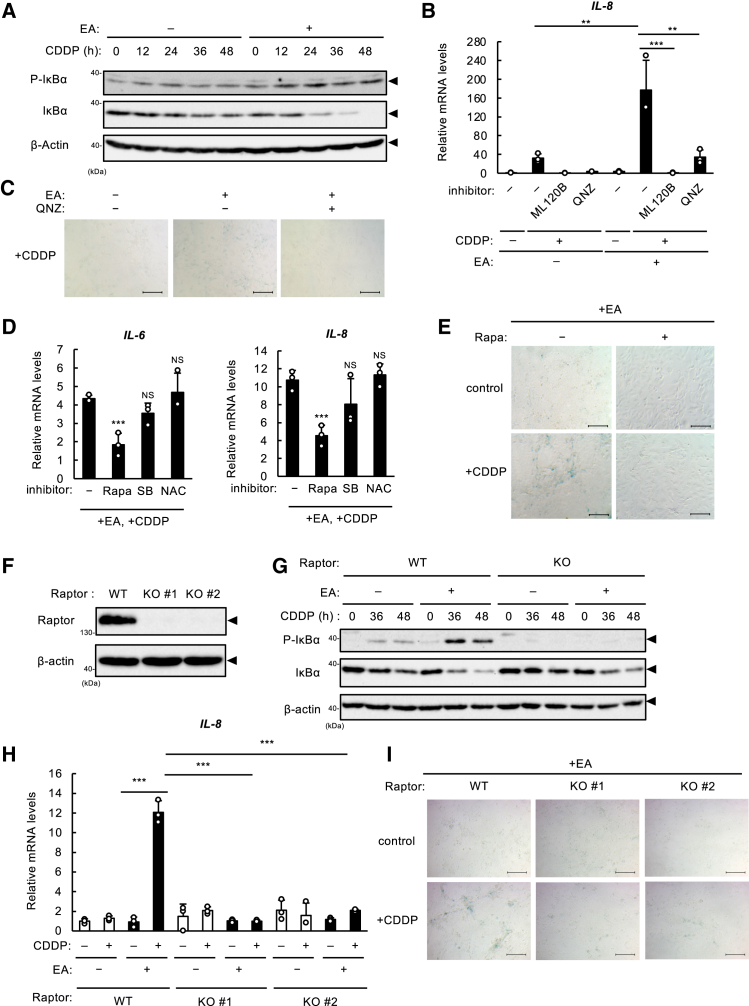
The mTORC1-NF-κB axis involves pro-senescence activity of EA (A) U2OS cells were pretreated with or without 200 μM EA for 12 h, and then stimulated with 10 μM CDDP for the indicated time periods. Cell lysates were subjected to immunoblotting with the indicated antibodies. (B) U2OS cells were pretreated with 200 μM EA for 12 h, treated with either IKK inhibitor ML120B (20 μM) or NF-κB inhibitor QNZ (5 μM) for 0.5 h, and then stimulated with 10 μM CDDP for 48 h, subjected to qRT-PCR analysis. Relative mRNA levels of *IL-8* are shown as mean ± SD (*n* = 3). (C) U2OS cells were pretreated with 200 μM EA for 12 h, treated with NF-κB inhibitor QNZ (5 μM) for 0.5 h, and then stimulated with 2 μM CDDP. After 48 h, the medium was replaced with a new medium with the same concentration of fatty acids and cultured for additional 5 days before being subjected to SA-β-gal staining. Scale bar, 100 μm (D) U2OS cells were pretreated with 200 μM EA for 12 h, treated with either mTOR inhibitor Rapamycin (Rapa 10 nM), p38 inhibitor SB203580 (SB, 5 μM) or antioxidant N-acetylcysteine (NAC, 1 mM) for 0.5 h, and then stimulated with 20 μM CDDP for 48 h, subjected to qRT-PCR analysis. Relative mRNA levels of *IL-6* and *IL-8* are shown as mean ± SD (*n* = 3). (E) U2OS cells were pretreated with 200 μM EA for 12 h, treated with mTOR inhibitor Rapamycin (Rapa 10 nM) for 0.5 h, and then stimulated with 2 μM CDDP. After 48 h, the medium was replaced with a new medium with the same concentration of fatty acids and cultured for 5 days. The cells were then subjected to SA-β-gal staining. Scale bar, 100 μm (F) *Raptor* WT and KO U2OS cells were lysed and subjected to immunoblotting with the antibodies against Raptor and β-actin. (G) *Raptor* WT and KO U2OS cells were pretreated with or without 200 μM EA for 12 h, and then stimulated with 10 μM CDDP for the indicated time periods. Cell lysates were subjected to immunoblotting with the indicated antibodies. (H) *Raptor* WT and KO U2OS cells were pretreated with or without 200 μM EA for 12 h, and then stimulated with 10 μM CDDP for 48 h, subjected to qRT-PCR analysis. Relative mRNA levels of *IL-8* are shown as mean ± SD (*n* = 3). (I) *Raptor* WT and KO U2OS cells were pretreated with 200 μM EA for 12 h, and then stimulated with 2 μM CDDP. After 48 h, the medium was replaced with a new medium with the same concentration of fatty acids and cultured for additional 5 days before being subjected to SA-β-gal staining. Scale bar, 100 μm. The images shown are from a representative sample (*n* = 1 of 3) from one of the three independent experiments. See also Figure S2.

To explore the molecular mechanism of pro-senescence effect of EA, we first tested whether EA sensitizes cells to DNA damage. As shown in Figures S2A and S2B, EA did not alter the level of phosphorylated histone H2A.X at serine 139 (γH2AX), a hallmark of DNA damage, upon DNA damage.^20^ We accordingly examined the involvement of p53, a master regulator of DNA damage response, including senescence.^15^ In p53 KO U2OS cells that we established in a previous report,^12^ p53 expression was not detected by immunoblot analysis with or without CDDP, confirming the depletion of p53 (Figure S2C). p53 deficiency blocked EA-mediated promotion of SA-β-gal-positive cell formation and IL-8 induction (Figures S2D and S2E; S5D); however, there was no significant effect of EA pretreatment on p53 protein levels in the nucleus and the mRNA level of p21, a representative downstream gene of p53, induced by CDDP (Figures S2F–S2G). These results suggested that while p53 is essential for CDDP-induced senescence and SASP, the enhancement of these responses by EA does not appear to involve the modulation of p53 activity. Of note, p21 protein levels were elevated in senescent cells treated with EA (Figure 1E), despite unchanged p21 mRNA levels (Figure S2G). This suggests the involvement of a p53-independent post-transcriptional mechanism – such as ubiquitination-dependent degradation – that regulates p21 abundance at the protein level.^21^^,^^22^^,^^23^ Thus, we focused on the NF-κB pathway which plays a major role in the induction of SASP factors during senescence.^15^ Under basal conditions, NF-κB activation is blocked due to its association with inhibitor of NF-κB α (IκBα). Upon ligand binding to receptors, such as Toll-like receptors (TLRs), IL-1Rs, and tumor necrosis factor (TNF) receptors, the downstream IκB kinase (IKK) complex is activated, resulting in the phosphorylation and subsequent degradation of IκBα. This degradation releases NF-κB, allowing its translocation into the nucleus, where it promotes the transcription of a wide variety of cytokines, including IL-6 and IL-8.^15^ To interrogate the mechanism underlying increased SASP gene expression by EA, we evaluated phophorylation status and expression levels of IκBα with or without EA. As shown in Figure 2A, EA pretreatment enhanced the phosphorylation of IκBα while reducing its protein expression over time after CDDP treatment, indicative of increased NF-κB activation. To address this possibility, we tested whether inhibitors for IKK and NF-κB, ML120B and 4-*N*-[2-(4-Phenoxyphenyl)ethyl]-1,2-dihydroquinazoline-4,6-diamine (referred to as QNZ), respectively, could disrupt the pro-senescence effect of EA. As anticipated, these inhibitors effectively reduced IL-8 mRNA levels and the number of SA-β-gal-positive cells elevated in the presence of EA (Figures 2B and 2C; S5E), supporting the involvement of the NF-κB pathway. Multiple molecules/factors have been associated with NF-κB activation during SASP, such as p38, ROS, and mammalian target of rapamycin (mTOR).^24^ In particular, we have previously shown that EA facilitates CDDP-induced ROS generation via NADPH oxidase and subsequent p38 activation, thereby promoting apoptosis.^12^ To determine which one is critical for SASP promotion by EA, we examined whether their inhibitors could reverse it. While a p38 inhibitor SB203580 (SB), an antioxidant N-acetylcysteine (NAC), a mitogen-activated extracellular signal-regulated kinase (MEK)/extracellular signal-regulated kinase (ERK) inhibitor (U0126) and a c-Jun N-terminal kinase (JNK) inhibitor (SP) were not effective in suppressing IL-6/8 overexpression by EA, an mTOR inhibitor rapamycin (Rapa) significantly suppressed it (Figures 2D and S2H). Rapa also reduced the increase in SA-β-gal-positive cells with EA pretreatment (Figure 2E; S5F), suggesting the contribution of mTOR to the pro-senescence activity of EA. There are two types of mTOR complexes, mTOR complex 1 (mTORC1) and mTORC2, which are distinguished by their specific components, regulatory protein associated with mTOR (Raptor) and rapamycin insensitive companion of mTOR (Rictor), respectively, governing various cellular processes including protein synthesis.^25^ Among these complexes, mTORC1 is the target of rapamycin,^25^ and has been shown to play a major role in regulating SASP by targeting IKK to augment its activation,^26^ and by promoting the translation of SASP-related molecules such as MAP kinase-activated protein kinase 2.^27^ To consolidate the results, we established Raptor KO U2OS cells by the Clustered Regularly Interspaced Short Palindromic Repeats (CRISPR)/CRISPR associated protein 9 (Cas9) system (Figure 2F), and evaluated the status of NF-κB activation in Raptor KO cells. Immunoblot analysis showed that the enhancement of IκBα phosphorylation-dependent degradation by EA, observed in WT cells, was suppressed in Raptor KO cells (Figure 2G). In agreement with this, EA-mediated increase in IL-8 mRNA levels and the number of SA-β-gal-positive cells were strongly inhibited by Raptor depletion (Figures 2H and 2I; S5G). These data suggest that the mTORC1-NF-κB axis participates in the pro-senescence activity of EA.

### Elaidic acid promotes SASP through the IL-1R-TAK1-NF-κB axis

**Figure 3:**
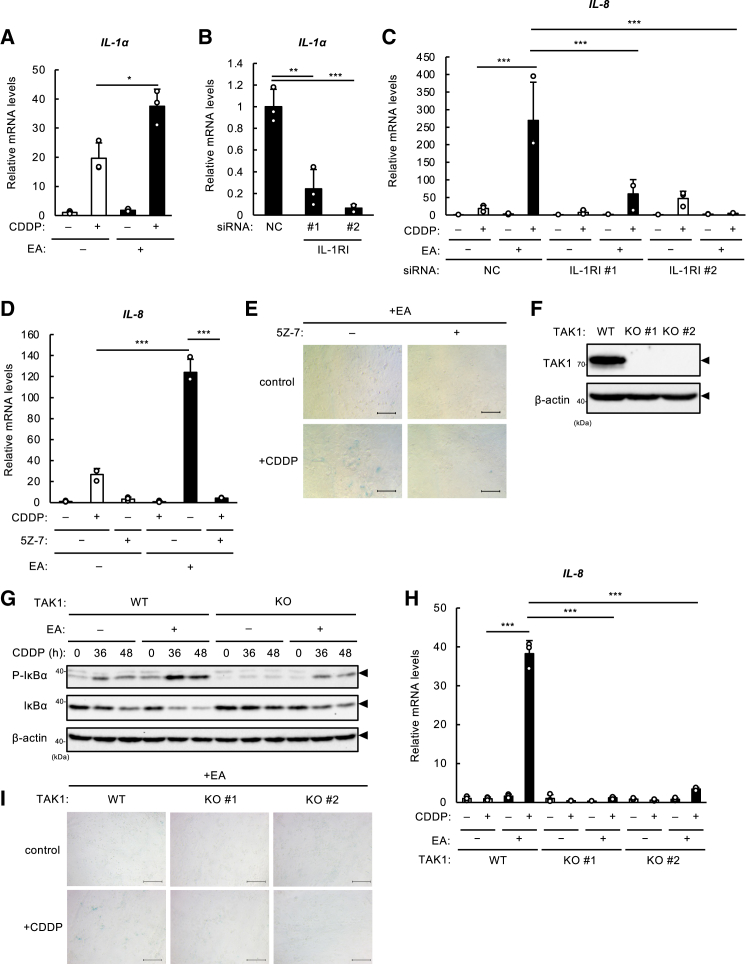
EA promotes SASP through the IL-1R-TAK1-NF-κB axis (A) U2OS cells were pretreated with or without 200 μM EA for 12 h, and then stimulated with 20 μM CDDP for 48 h, subjected to qRT-PCR analysis. Relative mRNA levels of *IL-1α* are shown as mean ± SD (*n* = 3). (B and C) U2OS cells were transfected with control or IL-1RI siRNA for 48 h, pretreated with 200 μM EA for 12 h, and then stimulated with 10 μM CDDP for 48 h, subjected to qRT-PCR analysis. Relative mRNA levels of *IL-1α* (b) and *IL-8* (c) are shown as mean ± SD (*n* = 3). NC: non-targeting control. (D) U2OS cells were pretreated with 200 μM EA for 12 h, treated with TAK1 inhibitor 5Z-7 (5 μM) for 0.5 h, and then stimulated with 10 μM CDDP for 48 h, subjected to qRT-PCR analysis. Relative mRNA levels of *IL-8* are shown as mean ± SD (*n* = 3). (E) U2OS cells were pretreated with 200 μM EA for 12 h, treated with TAK1 inhibitor 5Z-7 (5 μM) for 0.5 h, and then stimulated with 2 μM CDDP. After 48 h, the medium was replaced with a new medium with the same concentration of fatty acids and cultured for an additional 5 days before being subjected to SA-β-gal staining. Scale bar, 100 μm (F) *TAK1* WT and KO U2OS cells were lysed and subjected to immunoblotting with the antibodies against TAK1 and β-actin. (G) *TAK1* WT and KO U2OS cells were pretreated with or without 200 μM EA for 12 h, and then stimulated with 10 μM CDDP for the indicated time periods. Cell lysates were subjected to immunoblotting with the indicated antibodies. (H) *TAK1* WT and KO U2OS cells were pretreated with or without 200 μM EA for 12 h, and then stimulated with 10 μM CDDP for 48 h, subjected to qRT-PCR analysis. Relative mRNA levels of *IL-8* are shown as mean ± SD (*n* = 3). (I) *TAK1* WT and KO U2OS cells were pretreated with 200 μM EA for 12 h, and then stimulated with 2 μM CDDP. After 48 h, the medium was replaced with a new medium with the same concentration of fatty acids and cultured for an additional 5 days before being subjected to SA-β-gal staining. Scale bar, 100 μm. The images shown are from a representative sample (*n* = 1 of 3) from one of the three independent experiments.

One of the key activators of NF-κB during senescence is IL-1α, a representative SASP factor, that is a major translational target of mTOR signaling.^16^^,^^28^ Once IL-1α binds to IL-1R, mainly type I (referred to as IL-1RI), its adapter molecules, such as myeloid differentiation factor 88 (MyD88), IL-1 receptor-associated kinases (IRAKs), and TNF receptor-associated factor 6 (TRAF6), accumulate at the intracellular region, thereby activating a MAP kinase kinase kinase (MAP3K), TGF-β-activated kinase 1 (TAK1); TAK1 in turn phosphorylates IKKβ, leading to the activation of NF-κB^29^. NF-κB activation induces IL-1α expression and secretion, and amplifies IL-1R activation, constituting a feedforward activation loop of NF-κB, which results in further promotion of SASP and senescence.^16^ As we confirmed EA-dependent increase in IL-1α mRNA level upon CDDP treatment (Figure 3A) as well as IL-6/8 (Figures 1C and 1D), we knocked down *IL-1RI* in order to examine the role of IL-1R signaling (Figure 3B). As shown in Figure 3C, *IL-1RI-*konockdown remarkably suppressed CDDP-induced IL-8 overexpression by EA pretreatment, suggesting that IL-1R plays a crucial role in the EA-mediated amplification of SASP. To further verify this finding, we investigated whether TAK1 plays an essential role as well as IL-1R, using a TAK1 inhibitor 5Z-7-Oxozeaenol (5Z-7). As expected, 5Z-7 treatment effectively blocked the EA-mediated elevation of IL-8 mRNA levels and the number of SA-β-gal-positive cells, supporting the involvement of TAK1 (Figures 3D and 3E; S5H). Furthermore, we established TAK1 KO cells using the CRISPR/Cas9 system (Figure 3F), and found that TAK1 deficiency suppressed the enhancement of the phosphorylation and degradation of IκBα by EA pretreatment (Figure 3G). In consistent with this, TAK1 depletion also reversed the EA-mediated promotion of IL-8 induction and senescence upon CDDP treatment (Figures 3H and 3I; S5I). These results collectively suggest that EA promotes DNA damage-induced senescence via the IL-1R-TAK1-NF-κB axis.

### Elaidic acid preferentially accumulates in the lipid rafts, thereby potentiating the activation of the IL-1R signaling

**Figure 4:**
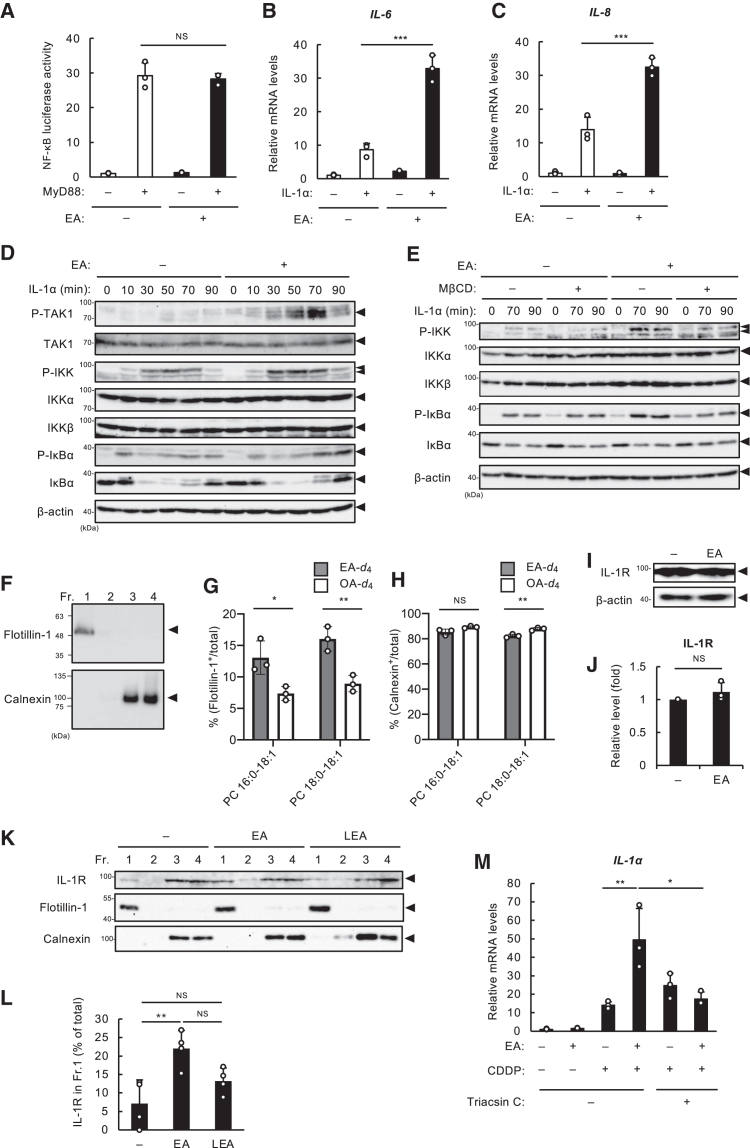
EA preferentially accumulates in the lipid rafts, thereby potentiating the activation of the IL-1R signaling (A) U2OS cells were pretreated with or without 200 μM EA for 12 h, and then overexpressed with Flag-MyD88 for 24 h, and assayed for NF-κB luciferase activity. Data are shown as mean ± SD (*n* = 3). (B and C) U2OS cells were pretreated with or without 200 μM EA for 12 h, and then stimulated with 10 ng/mL IL-1α for 12 h, subjected to qRT-PCR analysis. Relative mRNA levels of *IL-6* (b) and *IL-8* (c) are shown as mean ± SD (*n* = 3). (D) U2OS cells were pretreated with or without 200 μM EA for 12 h, and then stimulated with 10 ng/mL IL-1α for the indicated time periods. Cell lysates were subjected to immunoblotting with the indicated antibodies. (E) U2OS cells were pretreated with or without 200 μM EA for 12 h, treated with MβCD (5 mM) for 0.5 h, and then stimulated with 10 ng/mL IL-1α for the indicated time periods. Cell lysates were subjected to immunoblotting with the indicated antibodies. (F) Immunoblot analysis of DRM fraction. The four fractions from density gradient ultracentrifugation were analyzed by immunoblotting using anti-Flotillin-1 (DRM fraction corresponding to lipid rafts) and Calnexin (non-DRM fraction corresponding to endoplasmic reticulum, and so forth) antibody. (G and H) Ratios of tetra-deuterated EA and OA incorporated into phosphatidylcholine (PC) 16:0-18:1 and 18:0-18:1 in Flotillin-1- (g) and Calnexin- (h) positive fractions. Data are shown as mean ± SD (*n* = 3). (I and J) U2OS cells were treated with 200 μM EA for 12 h, and subjected to immunoblotting with the indicated antibodies (i). The band intensity of IL-R was normalized with that of β-actin, and shown as mean ± SD (*n* = 3) (j). (K and L) U2OS cells were treated with 200 μM EA or LEA for 12 h, followed by density gradient ultracentrifugation. The samples were separated into four fractions and analyzed by immunoblotting with the indicated antibodies (L). The band intensity of IL-R in Fr. 1 was quantified and expressed as a percentage of the total band intensity across all fractions. Data are represented as mean ± SD (*n* = 4). (M) U2OS cells were pretreated with 200 μM EA for 12 h, treated with ACSL inhibitor Triacsin C (0.5 μM), and then stimulated with 10 μM CDDP for 48 h, subjected to qRT-PCR analysis. Relative mRNA levels of *IL-8* are shown as mean ± SD (*n* = 3).

To determine the direct target of EA in the IL-1R-TAK1-NF-κB axis, we performed the NF-κB luciferase reporter gene assay to examine whether EA could promote NF-κB transcriptional activity upon the exogenous expression of MyD88, an adaptor molecule that directly interacts with IL-1R.^29^ We unexpectedly found that MyD88 expression induced NF-κB luciferase activity to the same level in the presence and absence of EA (Figure 4A), implicating that EA targets upstream of MyD88, namely IL-1R, to trigger the hyperactivation of the IL-1R-TAK1-NF-κB axis. To address this possibility, after EA pretreatment, U2OS cells were treated with recombinant IL-1α as a ligand of IL-1R, instead of CDDP. As shown in Figures 4B and 4C, EA significantly enhanced the induction of IL-6/8 mRNA expression upon IL-1α stimulation. In addition, immunoblot analysis showed that the IL-1α-induced phosphorylation of TAK1, IKK, and IκBα was facilitated by EA pretreatment (Figure 4D). Together, these data suggest that IL-1R becomes more sensitive to IL-1α stimulation in the presence of EA. Lipid rafts are heterogeneous microdomains in cell membranes, enriched in cholesterol, sphingolipids, and saturated acyl chains, serving as signaling platforms for various membrane receptors, such as B cell receptors.^30^ EA has been associated with lipid rafts, in which it facilitates the activation of epidermal growth factor receptors (EGFR), and promotes the expression of TLR4 and Fas receptor, thereby modulating pro-metastatic, inflammatory and cell death signaling, respectively.^31^^,^^32^^,^^33^ Since lipid rafts were also shown to be involved in IL-1R activation,^34^^,^^35^ we speculated that EA enriched in lipid rafts may play a role in the hyperactivation of IL-1R during DNA damage-induced senescence. To this end, we evaluated the activation of the NF-κB pathway upon IL-1α stimulation with or without methyl-β-cyclodextrin (MβCD), commonly used for removing cholesterol from the cell membrane to disrupt lipid rafts. As shown in Figure 4E, enhanced the phosphorylation of IKK-α/β and IκBα by EA pretreatment was suppressed in the presence of MβCD, supporting the notion that EA potentiates the activation of the IL-1R signaling in a manner dependent on lipid rafts. To determine the potential involvement of EA in lipid rafts, we performed a liquid chromatography-mass spectrometry/mass spectrometry (LC-MS/MS) analysis of detergent-resistant membranes (DRMs), corresponding to lipid rafts.^30^ Due to the identical molecular weights of EA and OA, conventional LC-MS/MS analysis cannot differentiate between these two isomers in phospholipids. To overcome this analytical hurdle, we employed EA deuterated specifically at the α- and β-positions (EA-*d*_4_)^36^ for LC-MS/MS analysis. DRMs were isolated by iodixanol density gradient ultracentrifugation, and subsequent immunoblot analysis revealed the successful DRM isolation in the top layer, light fraction with flotillin-1, a lipid raft marker (Figure 4F). Interestingly, LC-MS/MS analysis demonstrated that EA was incorporated into phosphatidylcholine (PC) species containing PA (C16:0) or stearic acid (SA, C18:0) at the *sn*1 position in lipid raft (DRM, Flotillin-1^+^) fraction (Fr. 1) compared to non-lipid raft (Calnexin^+^) fraction (Fr. 3 + 4), at a greater rate than OA (Figures 4G and 4H). To evaluate the relevance of this finding, we examined whether EA affects IL-1R protein levels in lipid rafts. While EA did not affect the IL-1R protein levels in whole cell lysates (Figures 4I and 4J), DRM fractionation followed by immunoblot analysis revealed that treatment with EA significantly elevated IL-1R protein levels in the lipid raft fraction (Fr.1) compared to control, an effect not observed with LEA (Figures 4K and 4l), one of the iTFAs which did not promote SASP (Figure S1D). These data suggest that EA has a specific capacity to promote IL-1R accumulation within lipid rafts, thereby facilitating the IL-1R signaling activation.^34^ Given the role of fatty acid acylation mediated by long-chain acyl-CoA synthetases (ACSLs) in membrane lipid synthesis, we utilized Triacsin C as an ACSL inhibitor, and found that Triacsin C treatment attenuated EA-dependent SASP promotion (Figure 4M), providing further support for the involvement of ACSL-mediated EA incorporation into lipid rafts and its subsequent contribution to SASP promotion.

### Elaidic acid promotes liver senescence and senescence-associated secretory phenotype in an high-fat diet mouse model

**Figure 5:**
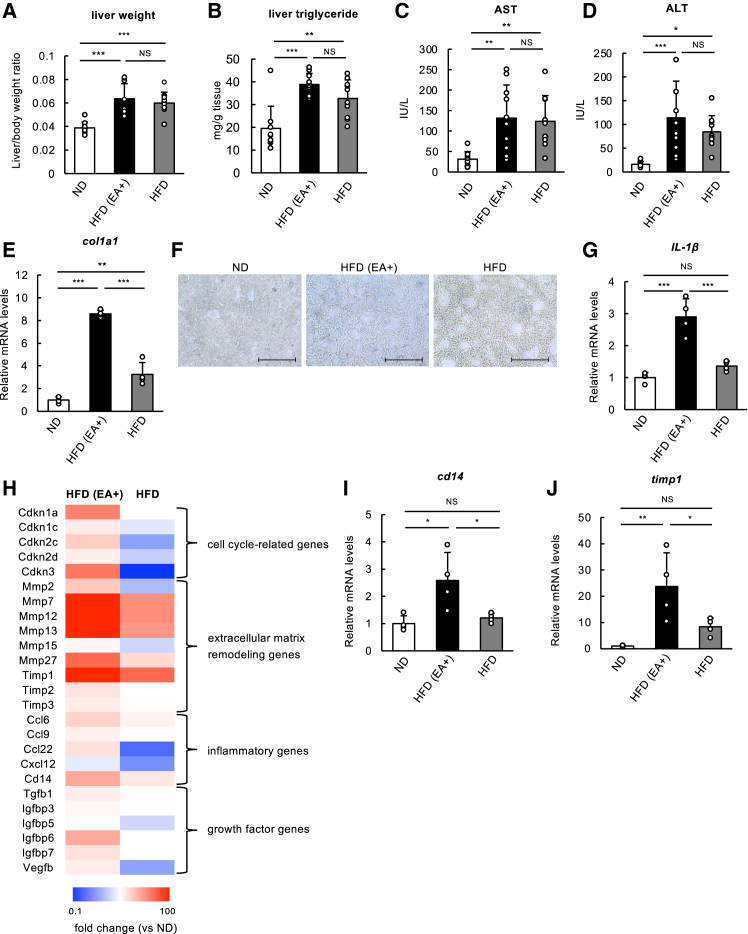
EA promotes liver senescence and SASP in an HFD mouse model (A and B) Liver was collected from mice fed each diet for 12 weeks, and used for measuring liver/body weight ratio (a) and liver triglyceride and (b) Data are shown as mean ± SD (*n* = 10). (C and D) Plasma from mice fed each diet for 12 weeks was used for measuring AST/ALT. Data are shown as mean ± SD (*n* = 10). (E) mRNA levels of *Col1A1* in livers from three groups were assessed by qRT-PCR. Data are shown as mean ± SD (*n* = 3) (F) Images of SA-β-gal staining of livers from mice fed each diet for 12 weeks. Scale bar, 100 μm (G) mRNA levels of *IL-1β* in livers from three groups were assessed by qRT-PCR. Data are shown as mean ± SD (*n* = 4). (H) Liver tissues of mice fed each diet for 12 weeks were subjected to RNA-seq analysis. mRNA levels of SASP factors that were upregulated in the presence of EA were selected and shown as fold changes in a heatmap. (I and J) mRNA levels of *Cd14*, *Timp1* in livers from three groups were assessed by qRT-PCR. Data are shown as mean ± SD (*n* = 4). The images shown are from a representative sample (*n* = 1 of 3) from one of the three independent experiments. See also Figure S3 and Tables S1–S3.

To extrapolate our findings to *in vivo* settings, we utilized a high-fat diet (HFD) mouse model to examine whether additional EA intake affects liver senescence and MASLD/MASH, which have been known as EA-related disorders.^2^ 8-week-old C57BL6/J male mice were randomly divided into three groups, and were fed the following three types of diets for 12 weeks: normal diet (ND) with 10% kcal fat, a diet with 40% kcal fat containing palm oil as normal HFD, or HFD with 40% kcal fat containing partially hydrogenated corn oil as HFD with EA (EA+) (Tables S1 and S2). By a gas chromatography-mass spectrometry (GC-MS) analysis, we confirmed that a substantial amount of EA was present in the liver only in HFD (EA+) group after 12 weeks of feeding (Table S3). Over the course of feeding, there was no difference in body weight and food intake between three groups (Figures S3A and S3B), but after 12 weeks, liver/body weight ratio, liver triglycerides, serum aspartate aminotransferase/alanine aminotransferase (AST/ALT) levels were significantly higher in mice fed HFD irrespective of the presence of EA (Figures 5A–5D). Of note, although mRNA level of collagen 1a1 (col1a1), a typical marker of liver fibrosis, was significantly upregulated in HFD (+EA) group compared to ND and HFD groups (Figure 5E), liver steatosis, neutral lipid accumulation and fibrosis – hallmark features of MAFLD/MASH – were observed at comparable levels in HFD groups with or without EA, as evidenced by Hematoxylin and Eosin (HE) staining, Oil Red O staining, and Sirius Red staining, respectively (Figures S3C–S3E). Nevertheless, intriguingly, the number of senescent cells, detected by SA-β-gal staining, was significantly higher in HFD (EA+) group compared to other two groups (Figure 5F; S5J), concomitant with the upregulation of p16, a representative senescence marker (Figures S3F and S3G), while in epididymal white adipose tissues, EA did not apparently affect the positivity of SA-β-gal staining (Figure S3H). These results suggest that EA consumption promotes senescence, particularly in the liver, during HFD-induced MASLD/MASH progression. In accordance with this result, mRNA levels of IL-1β, one of the representative SASP factors,^37^ were significantly upregulated only in the HFD (+EA) group (Figure 5G), suggestive of SASP promotion in the livers of mice fed the HFD (+EA) diet. To consolidate this notion, we performed an RNA-seq analysis of livers from three groups, and found a marked upregulation of genes associated with SASP in the HFD (EA+) group compared to other groups (Figures 5H, S3I, and S3J). To validate this result, we performed a quantitative real-time PCR (qRT-PCR) analysis. Indeed, a significant increase in mRNA levels of SASP factors, including a pro-inflammatory gene, CD14, and an extracellular matrix remodeling gene, TIMP Metallopeptidase Inhibitor 1 (Timp1), was observed only in the HFD (+EA) group (Figures 5I and 5J). Altogether, these data suggest that EA consumption promotes liver senescence and SASP in HFD-induced MASLD/MASH mouse model.

## Discussion

**Figure 6:**
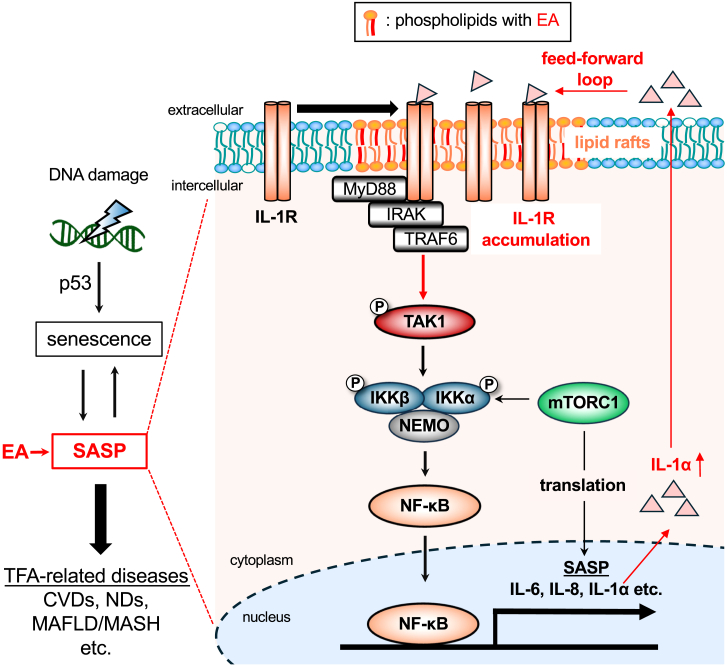
Schematic illustration of the mechanisms by which EA promotes cellular senescence and inflammation Upon DNA damage, EA, incorporated into lipid rafts as a phospholipid component, enhances IL-1R recruitment to the microdomains, thereby potentiating the activation of the IL-1R-TAK1-NF-κB axis, which leads to the promotion of SASP and cellular senescence. The secretion of SASP factors, such as IL-1α, initiates a positive feedback loop by further activating the IL-1R. This amplification of the inflammatory response ultimately contributes to the development and progression of TFA-related disorders, including CVDs, NDs, and MAFLD/MASH.

In this study, we demonstrated that EA, one of the most abundant TFAs in our body, promotes inflammatory responses by potentiating cellular senescence and SASP. EA amplified DNA damage-induced IL-1α signaling, leading to enhanced NF-κB activation and SASP factor production, ultimately promoting cellular senescence and inflammatory responses. These findings provide a mechanistic explanation for the pro-inflammatory effects of TFAs and their potential contribution to TFA-related diseases such as MASLD/MASH (Figure 6). We have recently revealed that iTFAs, namely EA and LEA, but not rTFAs, are potent promoters of apoptosis induced by eATP and DNA damage, although all of these TFAs were taken up by U2OS cells to a similar extent in the same experimental conditions.^13^^,^^14^ On the other hand, the pro-inflammatory effect characterized in this study was specific to EA, as other TFAs did not elicit a similar response. While ASK1, located in the cytosol, is a direct target of EA in response to eATP, and mitochondria serve as the primary target of EA in DNA damage-induced apoptosis, our findings suggest that the IL-1R within the plasma membrane lipid rafts is the primary target of EA in promoting inflammation during cellular senescence upon DNA damage. Given the varying target sites and mechanisms of action depending on the stress conditions, it would be reasonable that only EA exhibited a pro-inflammatory effect. However, the precise reason why EA alone exerts this effect remains unclear. The position of the double bond may influence the efficiency of acyl-CoA formation by ACSL and subsequent incorporation into lipid rafts. Further investigation is required to clarify the underlying mechanisms.

Lipid rafts are specialized microdomains within the plasma membrane that are enriched in saturated lipids, sphingolipids, and sterols, due to their strong intermolecular interactions,^30^ which provide a platform for the clustering of various receptors, such as T cell receptor, B cell receptor, TLRs and IL-1R, facilitating efficient signal transduction pathways.^30^ Notably, SFAs, including PA, have been shown to induce the dimerization and recruitment of TLRs, such as TLR-2/4/6, into lipid rafts, triggering their activation and subsequent inflammatory signaling, leading to NF-κB activation.^38^ In contrast, previous studies have shown that OA has anti-inflammatory effects by counteracting the pro-inflammatory actions of PA.^39^^,^^40^ To test whether the pro-inflammatory action of EA could be also mitigated by OA, we pretreated U2OS cells with EA together with OA, followed by CDDP treatment. As shown in Figures S4A and S4B, we unexpectedly found that OA could not reverse EA-mediated elevation of IL-8 mRNA levels, while suppressing IL-8 induction induced by PA. It has been widely believed that TFAs and SFAs exert similar biological effects; the linear molecular structure of EA facilitates tight molecular packing, resulting in similar physical properties to SA (C18:0), such as a solid state at room temperature and a higher melting point than OA, the geometrical isomer of EA.^41^ However, these collectively suggest that EA, localized within lipid rafts (Figures 4F–4H), may induce IL-1R activation via a distinct mechanism compared to SFAs, which is consistent with our earlier findings demonstrating the unique pro-apoptotic properties of iTFAs, including EA and LEA, compared to other fatty acids, including PA, during eATP and DNA damage.^9^^,^^10^ Interestingly, although LEA has been characterized as a pro-apoptotic TFA as well as EA,^9^^,^^10^ it did not exhibit any pro-inflammatory effect upon DNA damage, unlike EA, possibly due to more favorable accumulation of EA in lipid rafts than LEA, which in turn promoted IL-1R accumulation in these domains (Figures 4K and 4L).

Our *in vivo* experiments revealed that mice fed HFD with EA exhibited accelerated cellular senescence in comparison with those fed normal HFD without EA, particularly in the liver, as evidenced by increased SA-β-gal positivity (Figure 5F) and upregulated the expression of SASP factors such as IL-1β (Figures 5G–5J), suggesting a unique pro-inflammatory mechanism associated with EA. These findings indicate that EA can promote cellular senescence and inflammation, potentially contributing to the development and progression of MASLD/MASH. Although we observed an elevation of liver cellular senescence and SASP factor expressions (Figures 5F–5J), EA did not clearly exacerbate lipid accumulation or fibrosis in the liver (Figures 5B, S3C–S3E), likely due to the relatively short duration of the feeding period (12 weeks). Future studies are warranted to assess the long-term effects of EA consumption on liver senescence, inflammation, and the progression of MASLD/MASH. HFD and HFD (EA+) used in this study were made from different oils, palm oil and partially hydrogenated corn oil, respectively (Table S1); HFD (EA+) is enriched in EA, replacing ∼60% of SFAs (mainly PA) in HFD with EA, despite both diets being composed of the same amount of fat (40 kcal%) (Table S2). This gives rise to an implication that EA has a stronger pro-inflammatory and pro-senescence effect than SFAs in HFD-induced MASLD/MASH, although PA itself is much more pro-inflammatory than EA (Figures S4A and S4B). Intriguingly, as shown in Figure S4, while exogenous OA effectively suppressed IL-8 expression and IκBα degradation (i.e., NF-κB activation) induced by PA, it had no significant effect on these levels enhanced by EA in response to DNA damage. Considering that OA is a major fatty acid both in mouse diets and the body, as well as PA, PA-induced inflammation can be effectively mitigated by OA, whereas EA-induced inflammation remains unaffected and may even exacerbate with increased EA levels. Thus, in contrast to SFAs, which induce inflammation through well-characterized pathways involving NF-κB activation, EA appears to induce inflammation via NF-κB, but by a distinct mechanism, potentially involving lipid rafts and IL-1 signaling.

As our findings on the molecular mechanisms underlying EA-mediated pro-senescence and inflammatory effects were primarily based on experiments conducted in immortalized cancer cell lines, validating these findings in more physiologically relevant systems is of considerable importance. Primary hepatocytes or more complex *in vivo* models, such as those recapitulating MASLD/MASH, would offer valuable insight into the translational relevance of EA-mediated inflammation and senescence. Future investigations incorporating these systems will be crucial to determine the broader applicability of our mechanistic findings.

Previous studies have established a strong link between cellular senescence and various TFA-related diseases other than MASLD/MASH.^37^^,^^42^ Atherosclerosis, a major cause of CVDs, is characterized by the accumulation of plaques within arterial walls, which is initiated by the oxidation of LDL particles, leading to the recruitment and differentiation of monocytes into pro-inflammatory macrophages.^37^ Notably, senescent macrophages have been identified within atherosclerotic plaques from early stages of disease progression. As plaque formation advances, senescent vascular smooth muscle cells and endothelial cells further contribute to the disease by secreting pro-atherogenic factors such as IL-1α.^37^ Moreover, selective removal of senescent cells from atherosclerotic plaques has been shown to mitigate disease progression.^37^ Cellular senescence has also emerged as a critical factor in NDs, such as Alzheimer’s disease (AD).^42^ Senescent cells accumulate in the brains of aged mice and patients with AD, characterized by elevated levels of SASP factors such as IL-6.^42^ Importantly, senolytic therapies targeting senescent cells have demonstrated therapeutic potential in preclinical models of NDs.^42^ Given the pivotal role of cellular senescence in various diseases, our findings provide insights into the mechanisms by which TFAs, particularly EA, can induce cellular senescence and inflammation, underscoring the potential of targeting cellular senescence as a therapeutic strategy for preventing and treating TFA-related diseases. Pharmacological inhibition of IL-1R signaling, for example, with clinically approved IL-1R antagonists such as anakinra,^43^^,^^44^ may represent a promising strategy, since anakinra has demonstrated clinical benefit in several IL-1 driven conditions, including type 2 diabetes and atherosclerosis.^45^ Additionally, membrane-targeted approaches, including cholesterol-lowering agents (e.g., statins) and dietary interventions with omega-3 fatty acids (e.g., eicosapentaenoic acid (EPA) and docosahexaenoic acid (DHA)), may also provide complementary benefits by modulating the membrane microdomains that regulate signal transduction. Supplementation with omega-3 fatty acids has been shown to reduce inflammatory cytokines and CRP, a liver-derived marker of systemic inflammation.^46^ Notably, we have previously demonstrated that EPA and DHA effectively suppress the enhancement of extracellular ATP-induced apoptosis mediated by EA, which may be associated with ND and CVDs.^13^ These potential interventions warrant further investigation, particularly in the context of complex *in vivo* models of TFA-related diseases. Together, our findings highlight a mechanistic basis for the development of targeted therapeutic strategies aimed at ameliorating the inflammatory and senescence-associated consequences of dietary intake of TFAs such as EA.

### Limitations of the study

In this study, we found that EA is preferentially incorporated into phospholipids within lipid rafts, potentially driving IL-1R signaling activation by facilitating the recruitment of IL-1R to these microdomains, leading to enhanced cellular senescence and inflammation. However, the precise molecular mechanisms underlying these events remain to be clarified. A further limitation is that all mechanistic investigations were conducted using immortalized cancer cell lines, including U2OS, HepG2, and HeLa cells. While these models are well suited for dissecting signaling pathways, they may not fully recapitulate the physiological responses. In particular, the roles of IL-1R and lipid rafts in EA-mediated pro-senescence and pro-inflammatory activities warrant further validation under more physiologically relevant conditions, such as in primary culture cells or pathophysiological mouse models, including MASLD/MASH; genetically modified mice lacking IL-1R would help clarify the mechanistic contribution of IL-1R signaling within lipid rafts. These future studies will be essential to assess the broader applicability and translational significance of our findings.

## Resource availability

### Lead contact

Further information and requests for resources and reagents should be directed to and will be fulfilled by the lead contact, Yusuke Hirata (yusuke.hirata.d8@tohoku.ac.jp) or Atsushi Matsuzawa (atsushi.matsuzawa.c6@tohoku.ac.jp).

### Materials availability

All unique/stable reagents generated in this study are available from the lead contact upon request.

### Data and code availability

• The RNA-seq datasets generated in this study have been deposited in the DNA DataBank of Japan (DDBJ) under the accession number DRA019666. The data are publicly available at https://ddbj.nig.ac.jp/search/entry/sra-submission/DRA019666.
• No custom code was generated or used in this study.
• Any additional information required to reanalyze the data reported in this article is available from the lead contact upon request.

## Acknowledgments

We thank all members of the Lab of Health Chemistry for helpful discussions. This work was supported by JSPS/MEXT KAKENHI Grant Numbers JP20K07011, JP20KK0361, JP23K06111 (Y.H.), JP18J10828 (M.T), JP21H02620, JP21H00268, JP24K02173, JP24K22011 (A.M.), and by 10.13039/501100003478MHLW Grant Number JPMH23KA3004 (Y.H.). This work was also supported by the Japan Foundation for Aging and Health, Sapporo Bioscience Foundation, Lotte Research Promotion Grant, Mitsubishi Foundation, the Japan Foundation of Applied Enzymology, the Uehara Memorial Foundation, and the Takeda Science Foundation.

## Author contributions

Conceptualization and methodology: Y.H. and A.M.; investigation and formal analysis: R.K., Y.H., R.A., M.T., R.M., K.H., A.W., R.T., K.Y., T.N., and A.M.; data curation: R.K., Y.H., R.A., M.T., R.M., K.H., and A.M.; writing – original draft: R.K., Y.H. and A.M.; writing – review and editing: R.K., Y.H., and A.M.; funding acquisition: Y.H., and A.M.; resources: Y.H., K.H., A.W., R.T., E.S., T.A., K.Y., T.N., and A.M.; supervision: Y.H., and A.M.

## Declaration of interests

The authors declare that they have no conflicts of interest with the contents of this article.

## STAR★Methods

### Key resources table

**Table:** 

REAGENT or RESOURCE	SOURCE	IDENTIFIER
**Antibodies**		

Mouse monoclonal anti-p16	Cell Signaling	Cat#4824; RRID: AB_330138
Mouse monoclonal anti-p21	Cell Signaling	Cat#2946; RRID: AB_2260325
Mouse monoclonal anti-p53	Cell Signaling	Cat#2524; RRID: AB_331743
Mouse monoclonal anti-Raptor	Cell Signaling	Cat#2280; RRID: AB_561245
Rabbit polyclonal anti-phospho-IKKα/β	Cell Signaling	Cat#2697; RRID: AB_2079382
Mouse monoclonal anti-IKKα	Cell Signaling	Cat#2682; RRID: AB_331626
Mouse monoclonal anti-IKKβ	Cell Signaling	Cat# 8943; RRID: AB_11024092
Rabbit polyclonal anti-IκBα	Cell Signaling	Cat#9242; RRID: AB_331623
Mouse monoclonal anti-phospho-IκBα	Cell Signaling	Cat#9246; RRID: AB_2267145
Mouse monoclonal anti-TAK1	Cell Signaling	Cat#4505; RRID: AB_490858
Mouse monoclonal anti-phospho-TAK1	Thermo Fisher Scientific	Ca#MA5-15073; RRID: AB_10982333
Mouse monoclonal anti-flotillin-1	BD Biosciences	Cat#610820; RRID: AB_398139
Mouse monoclonal anti-β-actin	Santa Cruz	Cat#sc-47778; RRID: AB_626632
Rabbit polyclonal anti-calnexin	Santa Cruz	Cat#sc-11397; RRID: AB_2243890
Mouse monoclonal anti-laminA/C	Santa Cruz	Cat#sc-376248; RRID: AB_10991536
Rabbit polyclonal anti-IL-1R	Abcam	Cat#ab106278; RRID: AB_10865509
Mouse monoclonal anti-γH2AX	Santa Cruz	Cat#517348; RRID: AB_2783871
Rabbit polyclonal anti-tri-Me-K9-H3	Abcam	Cat#ab8898; RRID: AB_306848
Alexa Fluor 555-anti Mouse IgG	Invitrogen	Cat#A-21422; RRID: AB_2535844

**Chemicals, peptides, and recombinant proteins**		

Recombinant Interleukin-1α (IL-1α)	Biolegend	Cat#769702
Methyl-beta-cyclodextrin (MβCD)	Selleckchem	Cat#S6827
ML120B	Sigma	Cat#SML1174
SB203580 (SB)	Santa Cruz	Cat#sc-3533
5Z-7	Santa Cruz	Cat#sc-202055
Cisplatin (CDDP)	Wako	Cat#033-20091
N-acetyl-L-cystein (NAC)	Wako	Cat#017-05131
Rapamycin	LKT Laboratories	Cat#R0161
4-N-[2-(4-Phenoxyphenyl)ethyl]-1,2-dihydroquinazoline-4,6-diamine (QNZ)	Cayman	Cat#CAY10470
Triacsin C	Cayman	Cat#FR 900190
Palmitic acid (PA)	Nacalai Teque	Cat#25918-72
Oleic acid (OA)	Nacalai Teque	Cat#25630-64
Elaidic acid (EA)	Sigma	Cat#E4637
Lioellaidic acid (LEA)	Cayman	Cat#90160
Rumenic acid (RA)	Cayman	Cat#90140
Palmitoelaidic acid (PEA)	Cayman	Cat#9001798
*trans*-vaccenic acid (TVA)	Olbracht Serdary Research Laboratories	Cat#D-94
Fatty acid-free bovine serum albumin (BSA)	Wako	Cat#011-15144
H_2_O_2_	Wako	Cat#081-04215
1-Methyl-3-nitro-1-nitrosoguanidine (MNNG)	Tokyo Chemical Industry	Cat#M0527
U0126	Wako	Cat#211-01051
SP600125	Wako	Cat#197-16591
Lipofectamine RNAiMAX Transfection Reagent	Invitrogen	Cat#13778100
Triton-X100	Nacalai Tesque	Cat#35501-15
Fluoro-KEEPER Antifade Reagent	Nakalai Tesque	Cat#12593-64
protein assay CBB solution	Nakalai Tesque	Cat#29449-15
Luna Universal qPCR Master Mix	New England Biolabs	Cat#M3003
1% protease inhibitor cocktail	Nakalai Tesque	Cat#25955-11
Optiprep	Serumwerk Bernburg	Cat#1893
Normal diet (ND)	Research Diets	Cat#D09100304
high-fat diet (HFD) with EA	Research Diets	Cat#D16010101
HFD diet	Research Diets	Cat#D09100310N
O.C.T. compound	Sakura Finetek	Cat#45833
Mayer's Hematoxylin solution	Wako	Cat#131-09665
Eosin Y solution	Wako	Cat#058-00062
Entellan new	Sigma-Aldrich	Cat#1079610100
Oil Red O powder	Wako	Cat#154-02072
Sepasol	Nacalai Tesque	Cat#09379-55
Plasmocin	Invivogen	Cat#ant-mpt-1

**Critical commercial assays**		

Cellular Senescence Assay	Sigma	Cat#KAA002
Cell Titer 96 Cell Proliferation Assay	Promega	Cat#G5421
Dual-Luciferase® Reporter Assay System	Promega	Cat#E1910
Transaminase CII-test Wako	Wako	Cat#431-30901
Triglyceride E-test wako	Wako	Cat#291-94501
RNeasy Mini	Qiagen	Cat#74104

**Deposited data**		

DRA019666	DNA DataBank of Japan (DDBJ) database	https://ddbj.nig.ac.jp/search/entry/sra-submission/DRA019666

**Experimental models: Cell lines**		

Human U2OS (male)	ATCC	Cat#TIB-71
Human HeLa (male)	ATCC	Cat#CCL-121
Human HepG2 (male)	Cell Resource Center for Biomedical Research, Institute of Development, Aging and Cancer, Tohoku University	Cat#TKG 0205
HEK293T (female)	ATCC	Cat#CRL-11268
U2OS p53 KO #1	Hirata et al.,^12^	N/A
U2OS p53 KO #2	Hirata et al.,^12^	N/A
U2OS Raptor KO #1	This paper	N/A
U2OS Raptor KO #2	This paper	N/A
U2OS TAK1 KO #1	This paper	N/A
U2OS TAK1 KO #2	This paper	N/A

**Experimental models: Organisms/strains**		

Mouse: C57BL/6J	CLEA Japan	

**Oligonucleotides**		

See Table S4 for primers, siRNAs and gRNAs		
lentiCRISPRv2	Addgene^47^	Addgene Cat#52961; RRID: Addgene_52961
pCMV-VSV-G	Addgene	Addgene Cat#8454; RRID: Addgene_8454
psPAX2	Addgene	Addgene Cat#12260; RRID:Addgene_12260
MyD88	This paper	N/A
pGL4.32[luc2P NF-kB-RE Hygro]	Promega	Cat#E8491
pGL4.74[hRluc/TK]	Promega	Cat#E6921

**Software and algorithms**		

GraphPad Prism 9.5.1	GraphPad Software	https://www.graphpad.com/
ImageJ software (Fiji v. 2.3.0/1.53w)	NIH	https://imagej.net/
CytoExpert	Beckman Coulter	https://ls.beckmancoulter.co.jp/a/products/cytexpert/
iDEP.96	South Dakota State University	http://bioinformatics.sdstate.edu/idep96/

**Other**		

TLS55 centrifuge tubes	Beckman Coulter	Cat#347356
Zebron ZB-FAME 60m x 0.25mm x 0.20 μm	Phenomenex	Cat#7KG-G033-10
Select FAME 200 m × 0.25 mm	Agilent	Cat# CP7421
MAS-coated glass slides	Matsunami	Cat#S2215

### Experimental model and study participant details

#### Cell culture

The cells used in this study were cultured in a CO_2_ incubator at 37 °C with 5% CO_2_-95% air as the gas phase. U2OS cells, HepG2 cells and HeLa cells were maintained and passaged in Dulbecco’s Modified Eagle Medium (DMEM) (Nacalai Tesque, Kyoto, Japan) containing 5% FBS (U2OS, HeLa) or 10% FBS (HepG2) and 1% penicillin-streptomycin solution. The cell cultures were routinely treated with Plasmocin (#ant-mpt-1) (Invivogen, San Diego, CA USA) as a preventive measure against potential mycoplasma contamination.

#### Animal studies

Normal diet (ND) (#D09100304), high-fat diet (HFD) with EA (#D16010101) and HFD diet (#D09100310N) were purchased from Research Diets (New Brunswick, NJ USA). C57BL/6J mice (male, 7 weeks old) used in the study were purchased from CLEA Japan (Shizuoka, Japan). After acclimation for a week, mice were fed each diet *ad libitum* for 12 weeks, starting at 8 weeks of age. The ingredient composition of each feed is listed in Tables S1 and S2. Mice were maintained according to the Guidelines for Animal Experimentation of Tohoku University, and all the procedures were approved by the Institutional Animal Care and Use Committee at Tohoku University (approval number: 2018PhA-032 and 2020PhA-004).

### Method details

#### Preparation and treatment of fatty acids

Palmitic acid (PA, Cat#25918-72), oleic acid (OA, Cat#25702-82) (Nacalai Tesque), elaidic acid (EA, Cat#E4637, Sigma), linoeraidic acid (LEA, Cat#90160), rumenic acid (RA, Cat#90140), palmitoelaidic acid (PEA, Cat#9001798) (Cayman), *trans-*vaccenic acid (TVA, Cat#D-94) (Olbracht Serdary Research Laboratories, Etobicoke, Ontario, Canada) were each dissolved in 0.1 N NaOH at 70 °C and adjusted to 100 mM. Furthermore, fatty acid-free bovine serum albumin (BSA, Cat#013-15143, Wako, Tokyo, Japan) at pH 7.4 was allowed to react at 55°C for 10 minutes for conjugation to give a 5 mM fatty acid stock solution (10% BSA). When cells were treated with BSA-conjugated fatty acids, stock solution was diluted to a final concentration of 1% BSA in a medium with fetal bovine serum.

#### RNA extraction from cell lines

RNA extraction from cells was performed using Sepasol-RNA I (Nacalai Tesque). Cells seeded and stimulated in 24-well plates were washed with PBS and then lysed by adding 200 μL of Sepasol. 100 μL of chloroform/isoamylalcohol (50:1) was added to this and mixed well. After allowing to stand for 3 minutes, samples were centrifuged at 15,000 rpm and 4°C for 15 minutes, and the upper layer was transferred to another microtube. An equal volume of 2-propanol was added, followed by thorough mixing and a 10-minute incubation period. After centrifugation at 15,000 rpm and 4°C for 10 minutes, the precipitate was washed with 70% ethanol, air-dried, and dissolved in nuclease-free water. RNA concentration was calculated by measuring OD260.

#### Real-time PCR

Total RNA was extracted using Sepasol RNA I Super (Nacalai Tesque) and then was reverse transcribed into cDNA with High-Capacity cDNA Reverse Transcription Kit (Applied Biosystems, Waltham, MA, USA) according to the manufacturer’s instructions. mRNA levels were measured by qRT-PCR using Luna Universal qPCR Master Mix (New England Biolabs, Ipswich, MA, USA) and specific primers (Table S4), and normalized with those of *gapdh*.

#### Immunoblot assay

Cells were lysed in ice-cold lysis buffer containing 20 mM Tris–HCl, pH 7.4, 150 mM NaCl, 1% Triton-X100, 10% Glycerol, and 1% protease inhibitor cocktail (Cat#25955-11, Nacalai tesque). After centrifugation, the cell extracts were resolved by SDS-PAGE, and were analyzed as described previously.^48^ The antibodies used for immunoblotting were against, H3K9me3 (Cat#ab8898), IL-1R (Cat#ab106278), (Abcam, Cambridge, UK), p21 (Cat#2946), p53 (Cat#2524), Raptor (Cat#2280), phospho-IKKα/β (Cat#2697), IKKα (Cat#2682), IKKβ(Cat# 8943), IκBα (Cat#9242), phospho-IκBα (Cat#9246) and TAK1 (Cat#4505) (Cell Signaling, Danvers, MA, USA), phospho-TAK1 (Ca#MA5-15073) (Invitrogen, Waltham, MA, USA), Flotillin-1 (Cat# 610820) (BD Biosciences, Franklin Lakes, NJ, USA), β-actin (Cat#sc-47778), Calnexin (Cat#sc-11397) and lamin A/C (Cat#sc-376248) (Santa Cruz, Dallas, TX, USA). The blots were developed with ECL (Figure 4F, Takara, Shiga, Japan; the others, Merck Millipore, Burlington, MA, USA), and detected with Amersham Imager 680 (GE Healthcare, Chicago, IL, USA) in Figure 4F and ChemiDoc Touch Imaging System (BioRad, Hercules, CA, USA) in the other figures.

#### siRNA knockdown

siRNA targeting human *IL-1RⅠ* (Table S4) was obtained from GeneDesign (Osaka, Japan). U2OS cells were transfected with 10 nM non-targeting siRNA pool (Cat#D-001206-13, Dharmacon) as control or *IL-1RⅠ* siRNA using Lipofectamine RNAiMAX Transfection Reagent (Invitrogen), according to the manufacturer’s instructions.

#### Nuclear extraction

Nuclear extraction was performed as described previously.^49^ Cells were lysed in ice-cold lysis buffer containing 10 mM HEPES (pH 7.5), 10 mM KCl, 0.1 mM EGTA, 0.1 mM EDTA, 1 mM DTT, and 1% protease inhibitor cocktails (Cat#25955-11, Nacalai Tesque) for 15 min. Cell lysates were added 1% NP-40, and then centrifuged at 4 °C at 2,500 rpm for 3 min. After the supernatants, used as cytoplasmic fractions, were removed, the pellets were suspended in ice-cold lysis buffer containing 20 mM HEPES (pH 7.5), 400 mM NaCl, 1 mM EGTA, 1 mM DTT, and 1% protease inhibitor cocktails, and then vortexed every 5 min for 15 min. The resultant lysates were centrifuged at 4 °C at 15,000 rpm for 15 min, and then the supernatants were collected to be used as nuclear fractions.

#### Cell viability assay

U2OS cells were seeded on 96-well plates. After any stimulation or treatment, cell viability was determined using Cell Titer 96 Cell Proliferation Assay (Cat#G5421, Promega), according to the manufacturer’s protocol. The absorbance was read at 490 nm using a microplate reader (iMark, Bio-Rad). Data are normalized to control without stimulus, unless noted otherwise.

#### Immunocytochemistry

Immunocytochemistry was performed as described previously with minor modifications.^48^ U2OS cells were pretreated with or without 200 μM EA for 12 h, followed by treatment with 2 μM CDDP for 48 h. After CDDP treatment, the medium was replaced with fresh medium with or without EA, and cells were cultured for 5 days. Cells were fixed with 3.7% formaldehyde, permeabilized with 0.1% Triton-X100 (Cat#35501-15, Nakalai Tesque), blocked with 3% BSA-PBS, and incubated with primary antibodies overnight at 4 °C, followed by incubation with secondary antibodies with DAPI for 1 h at room temperature. The immunostained samples were enclosed with Fluoro-KEEPER Antifade Reagent (Cat#12593-64, Nakalai Tesque), and observed with a Zeiss LSM900 confocal fluorescence microscope. The antibodies used are as follows: mouse anti-γH2AX (#13989-1, Santa Cruz) (1:1000), and goat anti-mouse Alexa 555 (1:1000) (#A-21422, Invitrogen). Quantification was based on three images per sample, with data averaged from three independent samples and shown as mean ± SD (n = 3).

#### SA-β-gal staining for cell lines

SA-β-gal staining was performed using Cellular Senescence Assay (Cat#KAA002, Sigma-Aldrich) according to the manufacturer's recommended protocol. Cells were seeded on a 12-well plate, stimulated, washed with PBS, and fixed with Fixing Solution diluted 100-fold with PBS at room temperature for 15 minutes. After washing twice with PBS, SA-β-gal Detection Solution prepared by diluting Staining Solution A, B, and X-gal with PBS was added, and allowed to react overnight at 37°C in a moist and light-shielded environment. The cells were washed twice with PBS, stored with 70% glycerol/PBS, and observed with a microscope.

#### Generation of knockout cell lines

The CRISPR/Cas9 system^50^^,^^51^ was used to generate Raptor-, and TAK1-deficient cells. Guide RNA (gRNA) targeting each gene was designed using CRISPR direct^52^ (Table S4), and cloned into the lentiCRISPRv2 plasmid^47^ (obtained from Addgene, Cat#52961) and transfected into HEK293T (ATCC, Cat# ) cells together with the packaging plasmid Pax2 (Cat#12260) and the envelope plasmid pVSV-G (Cat#8454) (Addgene). After infecting U2OS cells with the recovered supernatant and selecting for puromycin resistance, cells were cloned by limiting dilution. Genomic sequences near the target region were amplified by PCR using the DNA extracted from each clone as a template and specific primers (Table S4), and introduction of gene mutation was confirmed by sequence analysis, and also by immunoblot analysis using the antibodies against each gene product.

#### Luciferase assay

Human MyD88 cDNA was cloned into Flag-tagged pcDNA3.2 by PCR using HeLa cell cDNA library as a template. Plasmid transfection into cells was performed using Polyethylenimine Max (Cat#24765, Cosmo Bio). Luciferase assay was performed using Dual-Luciferase (Cat#E1910, Promega). U2OS cells were seeded on a 96-well plate, pretreated with EA for 12 h, transfected with 5×κB Firefly luciferase (pGL4.32[luc2P NF-kB-RE Hygro], Cat#E8491, Promega) and thymidine kinase Renilla luciferase (pGL4.74[hRluc/TK], Cat#E6921, Promega), and incubated for 24 h. After removing the culture supernatant, 20 μL of Passive Lysis buffer diluted with MilliQ was added and shaken for 15 min at room temperature to obtain a cell lysate. Luminescence was detected using a multimode microplate reader, SpectraMax (Beckman Coulter, Brea, CA, USA). Luciferase Assay Buffer II and Luciferase Assay Substrate were mixed in advance, and 25 μL of Luciferase Assay Reagent II was applied to each 96-well plate for luminescence detection. After that, 25 μL of Stop&Glo Reagent premixed with Stop&Glo buffer and Stop&Glo Substrate was applied to a 96-well plate for luminescence detection, and Renilla activity for internal control was measured. Luciferase activity/Renilla activity corrected by dividing Luciferase activity by Renilla activity was defined as NF-κB activity.

#### The isolation of detergent-resistant membrane

Tetra-deuterated EA and OA were dried up under gentle N_2_ stream and dissolved in water containing 15 mM methyl-β-cyclodextrin (Cat#S6827, Selleckchem) at a concentration of 1 mM as ligand solutions. Cells were seeded in 6-well plate, and were treated with ligand solution at a final concentration of 30 μM of deuterated fatty acids for 24 h. Detergent-resistant membrane (DRM) was isolated as described previously.^53^ Briefly, cells were washed with PBS and TNE (250 mM Tris-HCl, pH7.4, 750 mM NaCl and 10 mM EDTA), and harvested with TNE. After brief centrifugation, cell pellets were suspended with 200 μL of TNE containing protease inhibitors, homogenized with 25-gauge needles (25 strokes), and mixed with Triton X-100 at a final concentration of 1%. After incubation on ice for 30 minutes, cell homogenates were mixed with 400 μL of 60% iodixanol using Optiprep (Cat#1893, Serumwerk Bernburg, Bernburg, Germany), transferred to TLS55 centrifuge tubes (Cat#347356, Beckman Coulter, Brea, CA, USA), and overlaid with 1.2 mL of 30% iodixanol and 200 μL of TNE containing protease inhibitors. After ultracentrifugation at 259,000 *g* for 2 h, four fractions were collected from the top of the gradient and subjected to immunoblot and LC-MS/MS analyses.

#### LC-MS/MS analysis

The amount of phosphatidylcholine (PC) containing a tetra-deuterated fatty acyl moiety were analyzed as described previously.^54^ The values of mass to charge ratio (*m/z)* for the selected reaction monitoring transitions were 764.5 (quadrupole 1 (Q1)) and 184.0 (Q3) for PC 16:0-18:1-*d*_4_, and 792.5 (Q1) and 184.0 (Q3) for PC 18:0-18:1-*d*_4_, respectively.

#### GC-MS analysis

Lipid extraction and fatty acid derivatization were performed as described previously.^55^ Briefly, lipids were extracted by the Bligh and Dyer method,^56^ and methylated with 2.5% H_2_SO_4_ in methanol. The resulting fatty acid methyl esters were extracted with hexane and subjected to GC-MS analysis. GC–MS analysis was performed using a GCMS-QP2010 Plus (Shimadzu, Kyoto, Japan) equipped with Zebron ZB-FAME 60m x 0.25mm x 0.20 μm (Phenomenex, Torrance, CA, USA) (Figures S4C and S4E; Table S3) or Select FAME 200 m × 0.25 mm (Agilent, Santa Clara, CA, USA) (Figures S4D and S4F). The oven temperature program was set as follows: the initial temperature was 100 °C for 2 min, then raised to 280 °C at 15 °C/min, and held for 5 min. The injector and detector temperatures were set at 240 °C and 260ºC (Figures S4C, 4E); the initial temperature was 185°C for 60 min, then raised to 215°C at a rate of 3°C/min and held for 50 min, while the vaporization chamber, ion source, and interface temperatures were set at 250°C, 200°C, and 250°C, respectively (Figures S4D and S4F). Helium gas was used as carrier at 74 psi and split ratio was set at 1:20. The amount of intracellular fatty acids were calculated based on standard curves created from serial dilution of the respective fatty acids, which was normalized with the amount of extracted protein measured by Bradford method using protein assay CBB solution (#29449-15, Nakalai Tesque).

#### Assessment of liver damage

Blood drawn from inferior vena cava was collected with heparin, then centrifuged at 4°C, 15,000 rpm for 10 min, and the supernatant was used as plasma. The activity of alanine aminotransferase (ALT) or aspartate aminotransferase (AST) in plasma was measured by Transaminase CII-test Wako (431–30901, Wako).

#### SA-β-gal staining of mouse tissues

Frozen liver tissues were sectioned at a thickness of 15 μm using a cryostat and mounted on MAS-coated glass slides to prepare frozen sections. After drying at room temperature for more than 30 minutes, fixed by reacting with Fixing Solution diluted 100-fold with PBS at room temperature for 15 minutes. Subsequently, the sections were processed for SA-β-gal staining using the Cellular Senescence Assay (KAA002, Sigma-Aldrich) following the same procedure as cell staining. Images were taken by Leica DMLB. Images were taken from three fields per mouse and from four mice per sample for quantitative analysis, and the data were shown as mean±SD (Figure S5J). Adipose tissue samples were trimmed to a uniform size and stained with SA-β-gal solution.

#### Immunohistochemistry of liver tissues

Livers of mice was immersed in O.C.T. compound (Cat#45833, Sakura Finetek, Tokyo, Japan) and immediately cooled to -80°C and frozen. Frozen tissues were sectioned at a thickness of 8 μm using a cryostat and mounted on MAS-coated glass slides (Cat#S2215, Matsunami, Osaka, Japan) to prepare frozen sections. After drying at room temperature for more than 30 min, fixed by reacting with 4% paraformaldehyde. After washing twice with PBS, 0.1% Triton X-100/PBS was added for 10 min. After washing with PBS, 3% BSA/PBS was added for 1 h then incubated with primary antibody diluted to PBS in wet box for 1 h. After washing with PBS for 5 min, incubated with secondary antibodies at room temperature for 1 hour. After washing with PBS for 5 minutes, samples were sealed with Fluoro-KEEPER Antifade Reagent Non-Hardening Type with DAPI (#12593-64, Nacalai Tesque) on glass slides (Matsunami). Anti-p16 antibody (Cat#4824, Cell Signaling) were used as primary antibodies, and Alexa Fluor 555-anti Mouse IgG (Cat#A-21422) (Invitrogen) was used as secondary antibodies. Samples were observed with Zeiss LSM800 laser confocal microscope (Carl Zeiss). Images were taken from three fields per mouse and from three mice per sample for quantitative analysis, and the quantitative data were shown as mean±SD (Figure S3G).

#### HE staining

Paraffin block was sectioned at a thickness of 8 μm using microtome then mounted to MAS-coated glass slide (Matsunami) to prepare paraffin section. After washing with water, the cells were stained with Mayer's Hematoxylin solution (Cat#131-09665, Wako) for 10 min; after 10 min of color removal with water, the cells were stained with Eosin Y solution (Cat#058-00062, Wako) for 2 min. After washing and dehydration, tissues were sealed in Entellan new (Cat#1079610100, Sigma-Aldrich).

#### Sirius red staining

Paraffin section was prepared as described above. After washing, sections were fixed with Bouin solution containing 0.0088 % formalin, 4.8% acetic acid and 71% saturated picric acid at 55°C for 1 h, and then incubated with 1% acetic acid for 2 min. Afterwards, tissues were incubated with 0.1% Sirius red for 30 min. After washing and dehydration, tissues were sealed in Entellan neu (Cat#1079610100, Sigma-Aldrich ).

#### Triglyceride measurement

Triglyceride in mouse livers was measured using Triglyceride E-test wako (Cat#291-94501, Wako industry), according to the manufacture’s protocol. Livers of mice were homogenized with 2 times the liver weight (v/w) of homogenization buffer containing 75 mM KPi-KCl (pH7.4). Homogenate was added to 3 times of liver weight of extraction buffer containing 75mM KPi-KCl (pH7.4) and 2% Triton X-100, then incubated for 10 min and heated at 65°C for 30 min. Triglyceride was measured using supernatant centrifuged at 15,000 rpm for 10 minutes.

#### Oil Red O staining

Frozen liver tissues of mice were sectioned at 8 μm using cryostat and mounted to MAS-coated glass slide (Matsunami) to prepare a frozen section. Frozen section was incubated with 60% isopropyl alcohol at room temperature for 5 min then stained with Oil Red O (ORO) working solution at room temperature for 15 min. Glass slide was incubated with 60% isopropyl alcohol at room temperature for 2 min, then washed with water and stained with Mayer’s Hematoxylin (Cat#131-09665, Wako) at room temperature for 30 sec. 70% glycerol was used for sealing. To prepare the ORO working solution, first, 2.5 grams of Oil Red O powder (Cat#154-02072, Wako) was dissolved in 400 mL of isopropyl alcohol, and the mixture was stirred continuously at room temperature for at least 2 h. Once the Oil Red O powder was completely dissolved, the stock solution was diluted by mixing three parts of the stock solution with two parts of water. The diluted solution was let stand at 4ºC for 5-10 min, and filtered through Whatman filter paper to remove any undissolved particles.

#### Protein extraction from liver tissues

Liver tissues were trimmed, and protein was extracted using 400 μL of RIPA lysis buffer. Lysate was centrifuged at 12,000 × *g*, 4ºC for 10 minutes and the supernatant was centrifuged at 15,000 rpm, 4ºC for 15 minutes. Samples were prepared mixing 150 μL supernatant and 30 μL 6 × SB, and were used for immunoblot analysis.

#### RNA extraction from liver tissues

Liver tissues were trimmed, and homogenized in 500 μl of Sepasol (Cat#09379-55, Nacalai Tesque) using a polytron. After centrifugation at 12,000 × *g*, 4ºC for 10 min, the supernatant was then mixed with 100 μL of CIA and RNA was subsequently extracted as described above, in RNA extraction from cell lines.

#### RNA-seq analysis

RNA was purified from mouse livers fed each diet for 12 weeks using RNeasy Mini (Cat#74104, Qiagen, Hilden, Germany) according to the manufacture’s protocol, and RNA-seq analysis was performed by Bioengineering Lab (Kanagawa, Japan). The data obtained was analyzed using iDEP.96 (http://bioinformatics.sdstate.edu/idep96/). 6,000 most variable genes were clustered into six groups. For each group, enriched pathways were identified using the Gene Ontology Biological Process database.

### Quantification and statistical analysis

#### Statistics

All data are presented as the mean ± standard deviation (SD). All experiments were repeated at least three independent times. Statistical analysis was performed using GraphPad Prism software (version 9.5.1). Two-group comparisons were analyzed by two-tailed Student's t-test, while multiple-group comparisons were analyzed by one-way or two-way ANOVA followed by Tukey's or Dunnett's test. Statistical significance was determined versus control unless otherwise noted. Values of ∗p < 0.05, ∗∗p < 0.01, and ∗∗∗p < 0.001 were considered statistically significant. NS, not significant.
