# Supplementary material for: Elaidic acid drives cellular senescence and inflammation via lipid raft-mediated IL-1R signaling
Source: iScience. 2025 Aug 6;28(9):113305. doi: 10.1016/j.isci.2025.113305 (PMC12396248; doi:10.1016/j.isci.2025.113305)
Supplement: Document S1. Figures S1–S6 and Tables S1–S4 [file mmc1.pdf]

iScience, Volume *28*

Supplemental information

Elaidic acid drives cellular senescence and inflammation via lipid raft-mediated IL-1R signaling

Ryota Kojima, Yusuke Hirata, Ryo Ashida, Miki Takahashi, Ryosuke Matsui, Kotaro Hama, Ayako Watanabe, Ryo Takita, Emiko Sato, Taiki Abe, Kazuaki Yokoyama, Takuya Noguchi, and Atsushi Matsuzawa

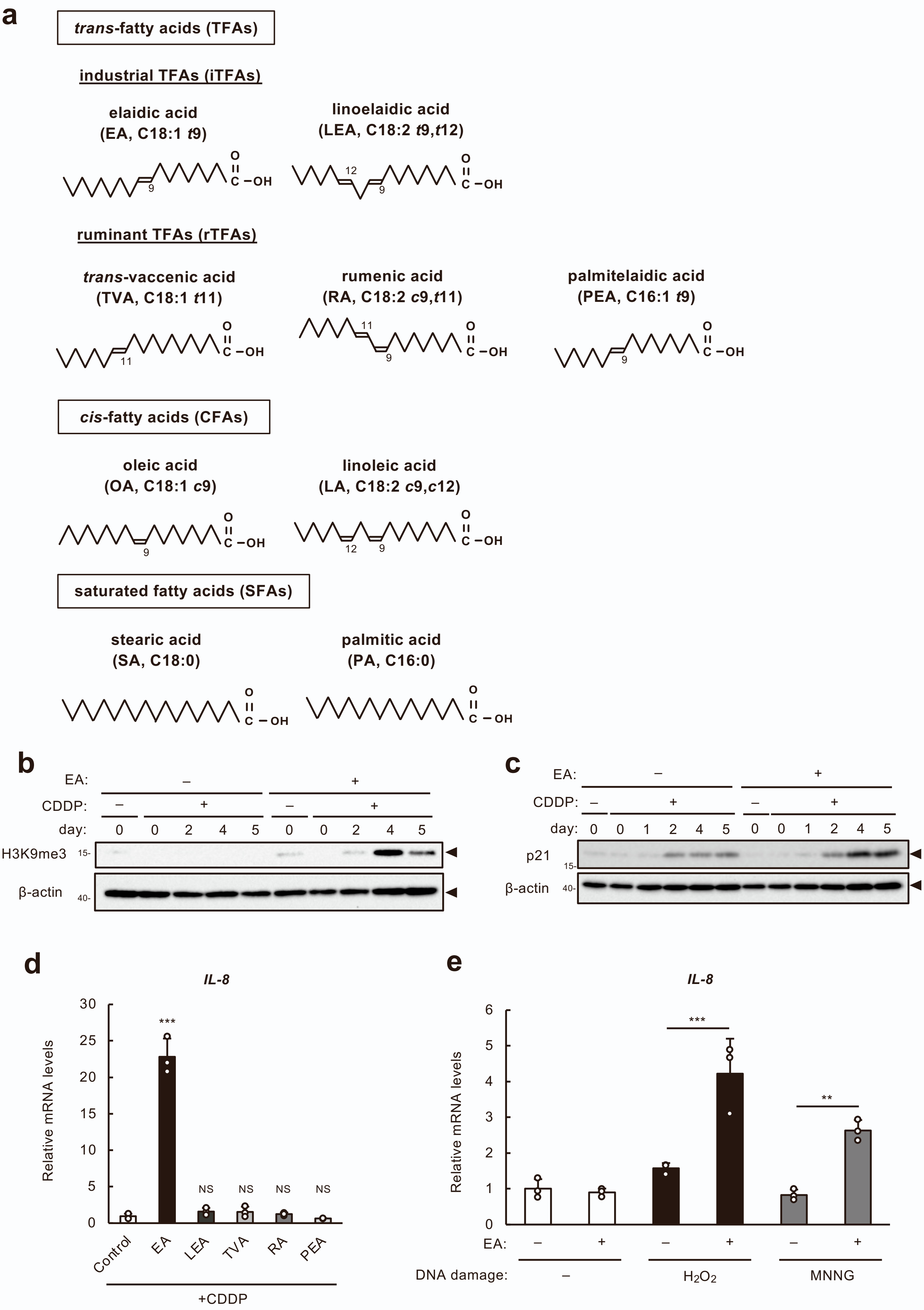

Kojima et al., Fig. S1

Supplementary figure 1. EA facilitates IL-8 induction in response to DNA damage, related to Figure 1

(a) The structures of fatty acids presented in this paper. In parentheses, the following information is provided in order: abbreviation, the number of carbon atoms, configuration (*t*, *trans*; *c*, *cis*) and positions of the carbon-carbon double bonds. (b, c) U2OS cells were pretreated with 200 μM EA for 12 h, followed by treatment with 2 μM cisplatin for 48 h. After cisplatin treatment, the medium was replaced with fresh medium containing EA but without cisplatin, and cells were cultured for an additional 5 days. Cell lysates were subjected to immunoblotting with the indicated antibodies at the indicated time points. (d) U2OS cells were pretreated with the indicated TFAs at 200 μM for 12 h, and then stimulated with 10 μM CDDP for 48 h, subjected to qRT-PCR analysis. Relative mRNA levels of *IL-8* are shown as mean ± SD (n = 3). (e) U2OS cells were pretreated with or without EA at 200 μM for 12 h, and then stimulated with H_2O_2 (100 μM) or MNNG (20 μM) for 48 h, subjected to qRT-PCR analysis. Relative mRNA levels of *IL-8* are shown as mean ± SD (n = 3).

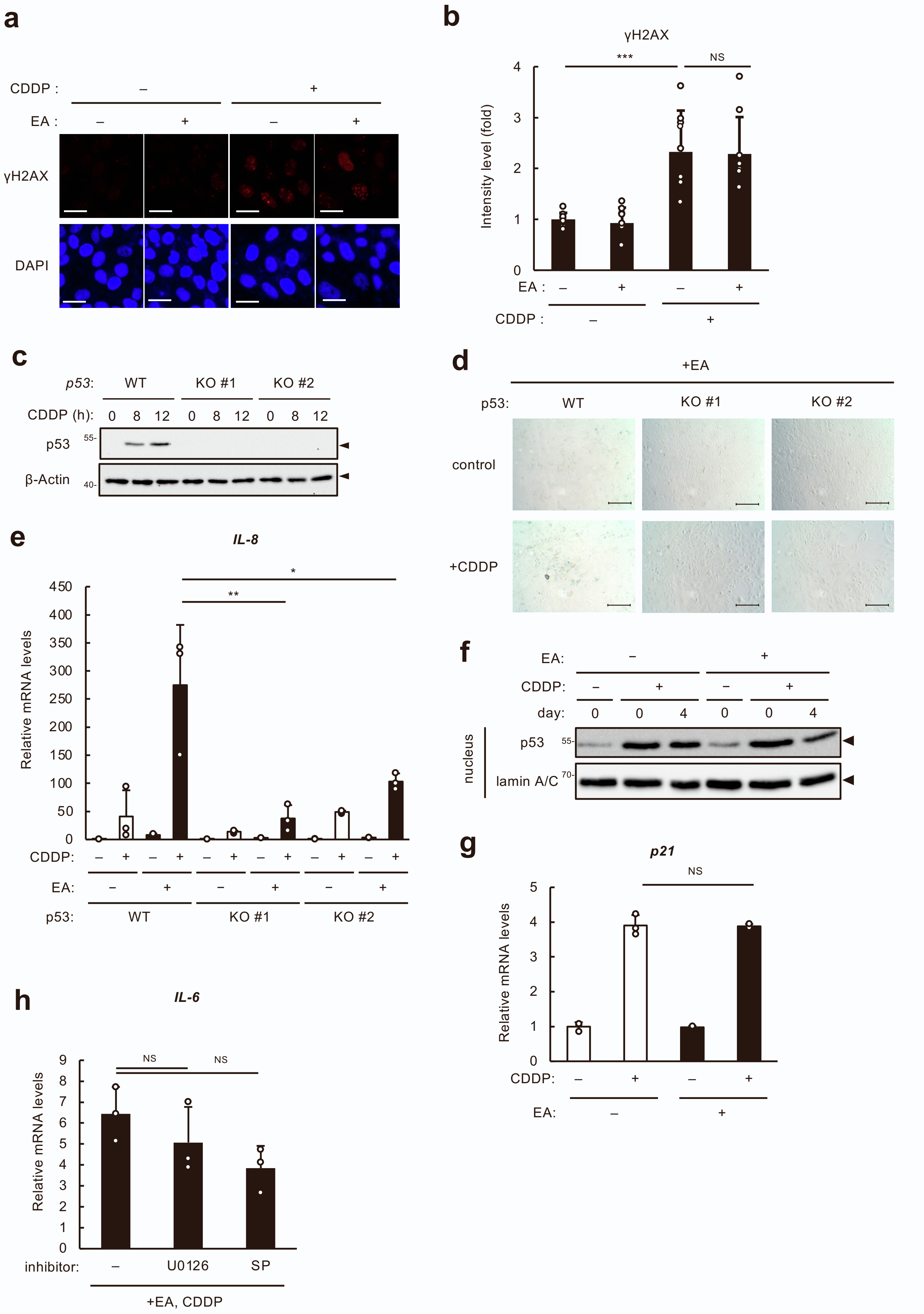

Kojima et al., Fig. S2

Supplementary figure 2. EA promotes DNA damage-induced senescence in a p53-dependent manner without affecting its expression or activity, related to Figure 2

(a, b) Representative data of immunocytochemistry for γH2AX in U2OS cells. Scale bar, 100 µm (a). For quantification of γH2AX signal intensity, three fields from each of three independent samples were analyzed. Data are shown as mean ± SD (n = 9). (c) *p53* WT and KO U2OS cells were treated with 40 µM CDDP for the indicated time periods. Cell lysates were subjected to immunoblotting with the indicated antibodies. (d) *p53* WT and KO U2OS cells were pretreated with 200 µM EA for 12 h, and then stimulated with 2 µM CDDP. After 48 h, the medium was replaced with a new medium with the same concentration of fatty acids and cultured for additional 5 days before being subjected to SA-β-gal staining. Scale bar, 100 µm. (e) *p53* WT and KO U2OS cells were pretreated with or without 200 µM EA for 12 h, and then stimulated with 20 µM CDDP for 48 h, subjected to qRT-PCR analysis. Relative mRNA levels of *IL-8* are shown as mean ± SD (n = 3). (f) U2OS cells were pretreated with or without 200 µM EA for 12 h, and then stimulated with 10 µM CDDP. After 48 h, the medium was replaced with a new medium with the same concentration of fatty acids and cultured for the indicated time periods. Nuclear extracts obtained from the cell lysates were subjected to immunoblotting with the indicated antibodies. (g) U2OS cells were pretreated with or without 200 µM EA for 12 h, and then stimulated with 10 µM CDDP for 48 h, subjected to qRT-PCR analysis. Relative mRNA levels of *p21* are shown as mean ± SD (n = 3). (h) U2OS cells were pretreated with 200 µM EA for 12 h, treated with either a MEK/ERK inhibitor U0126 (20 µM), a JNK inhibitor SP600125 (SP, 5 µM), and then stimulated with 20 µM CDDP for 48 h, subjected to qRT-PCR analysis. Relative mRNA levels of *IL-6* are shown as mean ± SD (n = 3).

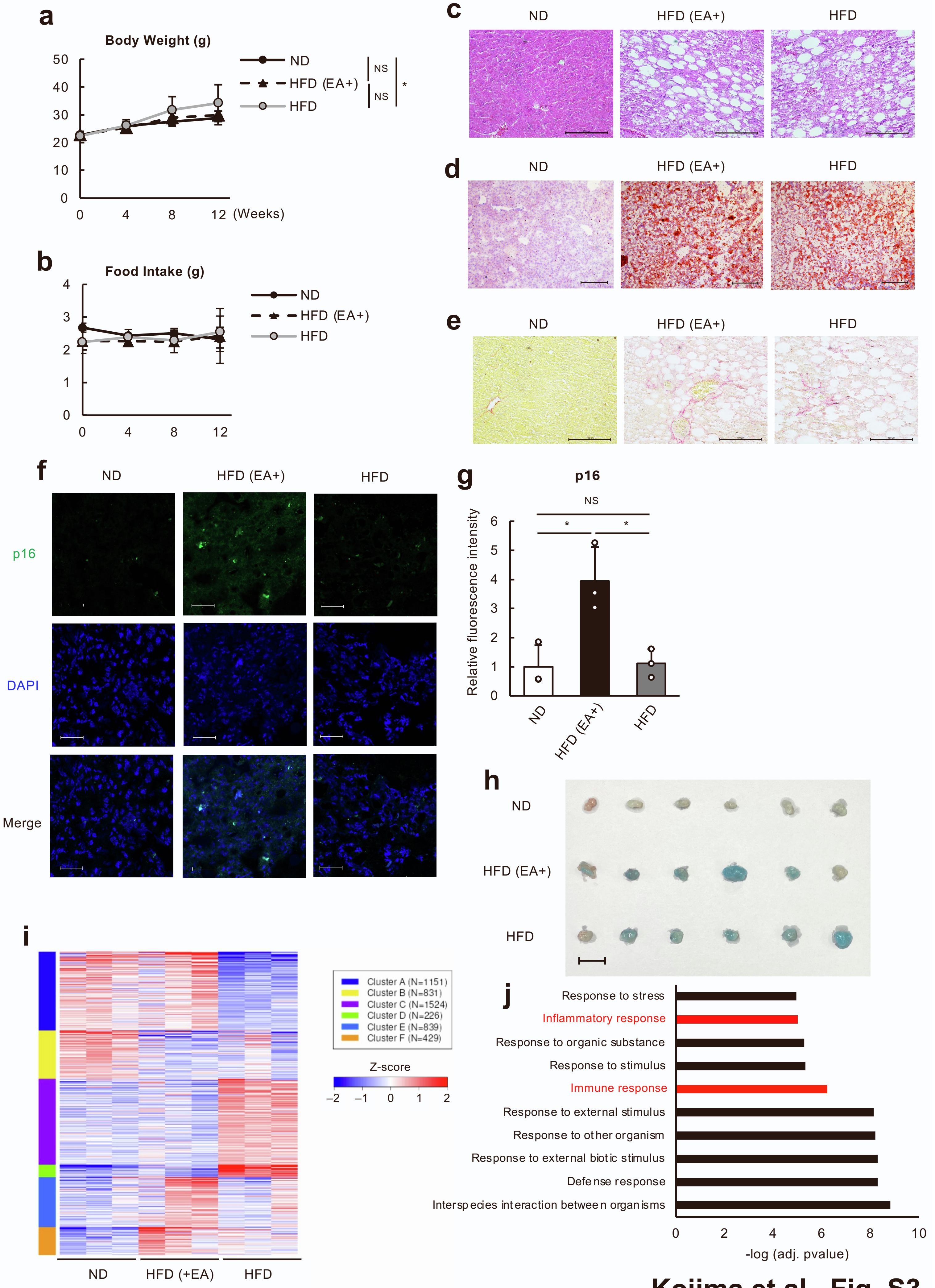

Kojima et al., Fig. S3

Supplementary figure 3. EA intake promotes liver SASP and inflammation in the HFD mouse model, related to Figure 5

(a, b) Over a 12-week period, the body weight (a) and food intake (b) of mice were measured. Data are shown as mean ± SD (n = 20). (c-g) HE staining (c), Oil red O staining (d), Sirius red staining (e), and immunohistochemistry for p16 (f, g) of liver sections from three groups after 12-week feeding period. For quantification of p16 signal intensity in (f), the average of three images was calculated for each mouse, and the average of three mice per group was determined and shown as mean ± SD (n = 3) Scale bar, 100 µm. (g). (h) SA-β-gal staining of epididymal white adipose from three groups fed each diet for 12 weeks. Scale bar, 10 mm. (i-j), K-means clustering analysis was performed on RNA-seq data from the livers of mice fed each diet for 12 weeks (i). Significantly enriched pathways in cluster F, with SASP-related pathways indicated in red (j).

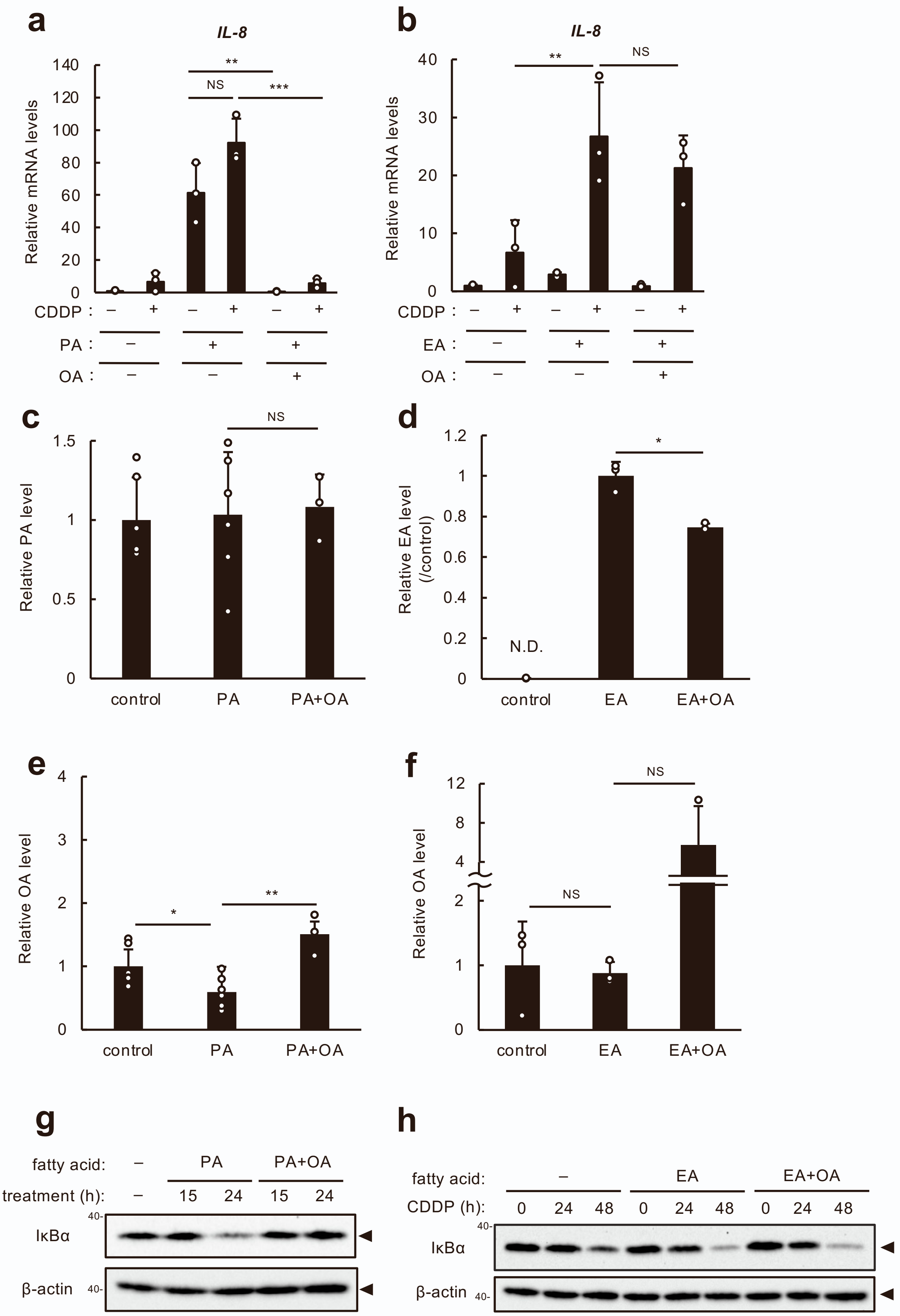

Kojima et al., Fig. S4

Supplementary figure 4. The pro-inflammatory effect of EA was not reversed by OA, unlike the effect of PA, related to Discussion

(a, b) U2OS cells were pretreated with indicated fatty acids for 12 h, and then stimulated with 10 µM CDDP for 48 h, subjected to qRT-PCR analysis. Relative mRNA levels of *IL-8* are shown as mean ± SD (n = 3). (c-f) U2OS cells were treated with the indicated fatty acids for 12 h. Lipids were extracted from cells, derivatized by methylation, and then subjected to GC-MS analysis. Relative molar amounts of incorporated PA (c), EA (d), and OA (e, f) are shown as mean ± SD (e; control, PA n=6 , others; n=3). (g) U2OS cells were treated with the indicated fatty acids for indicated time periods. Cell lysates were subjected to immunoblotting with the indicated antibodies. (h) U2OS cells were pretreated with the indicated fatty acids for 12 h, and then stimulated with 10 µM CDDP for the indicated time periods. Cell lysates were subjected to immunoblotting with the indicated antibodies.

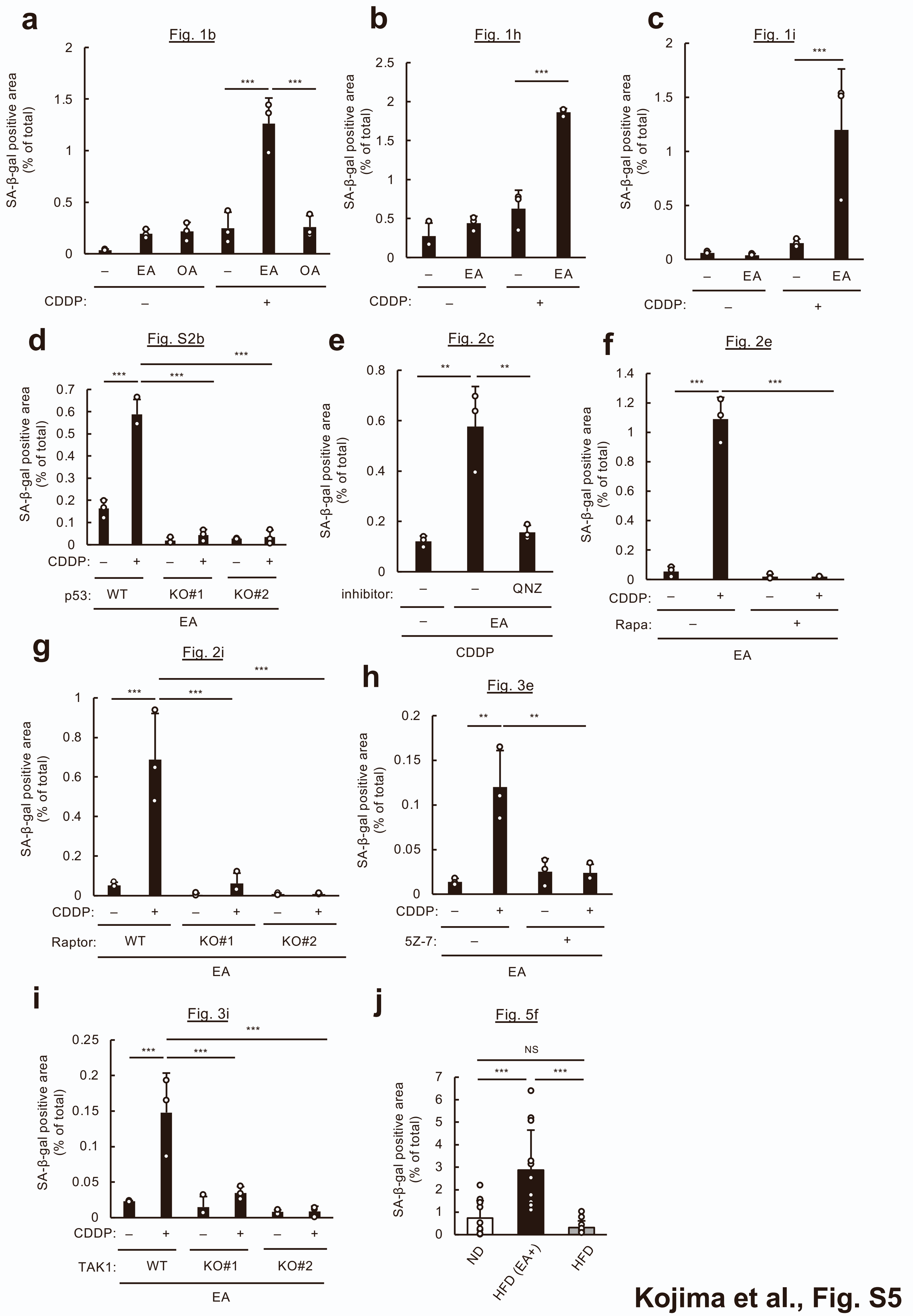

Kojima et al., Fig. S5

Supplementary figure 5. Quantification of SA-β-gal staining data

(a-k) Quantified SA-β-gal positive area are shown as mean ± SD (j, n=9; others, n=3): Fig. 1b (a), Fig. 1h (b) , Fig. 1i (c), Fig. S2b (d), Fig. 2c (e), Fig. 2e (f), Fig. 2i (g), Fig. 3e (h), Fig. 5i (i), Fig. 5f (j).

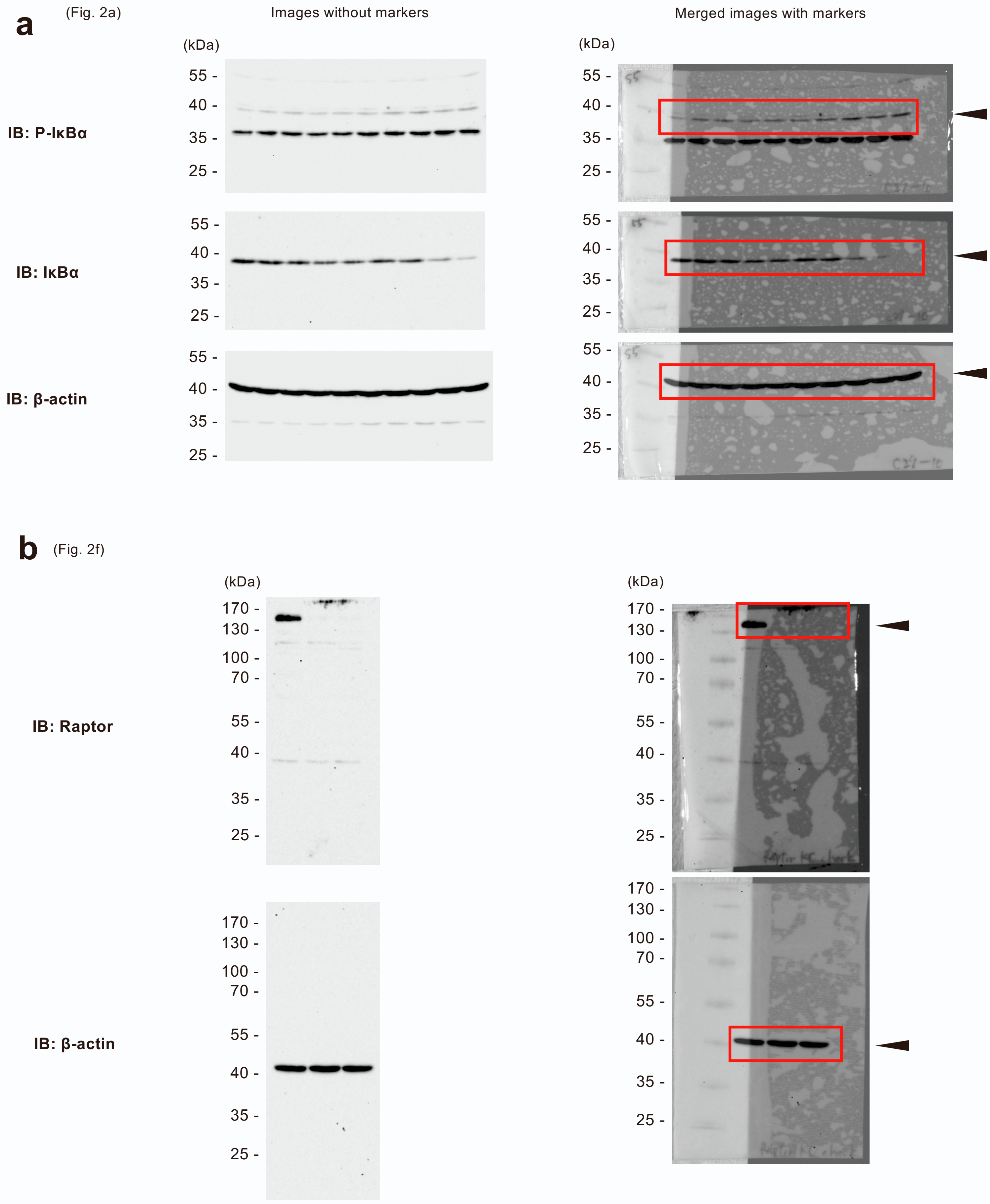

Kojima et al., Fig. S6

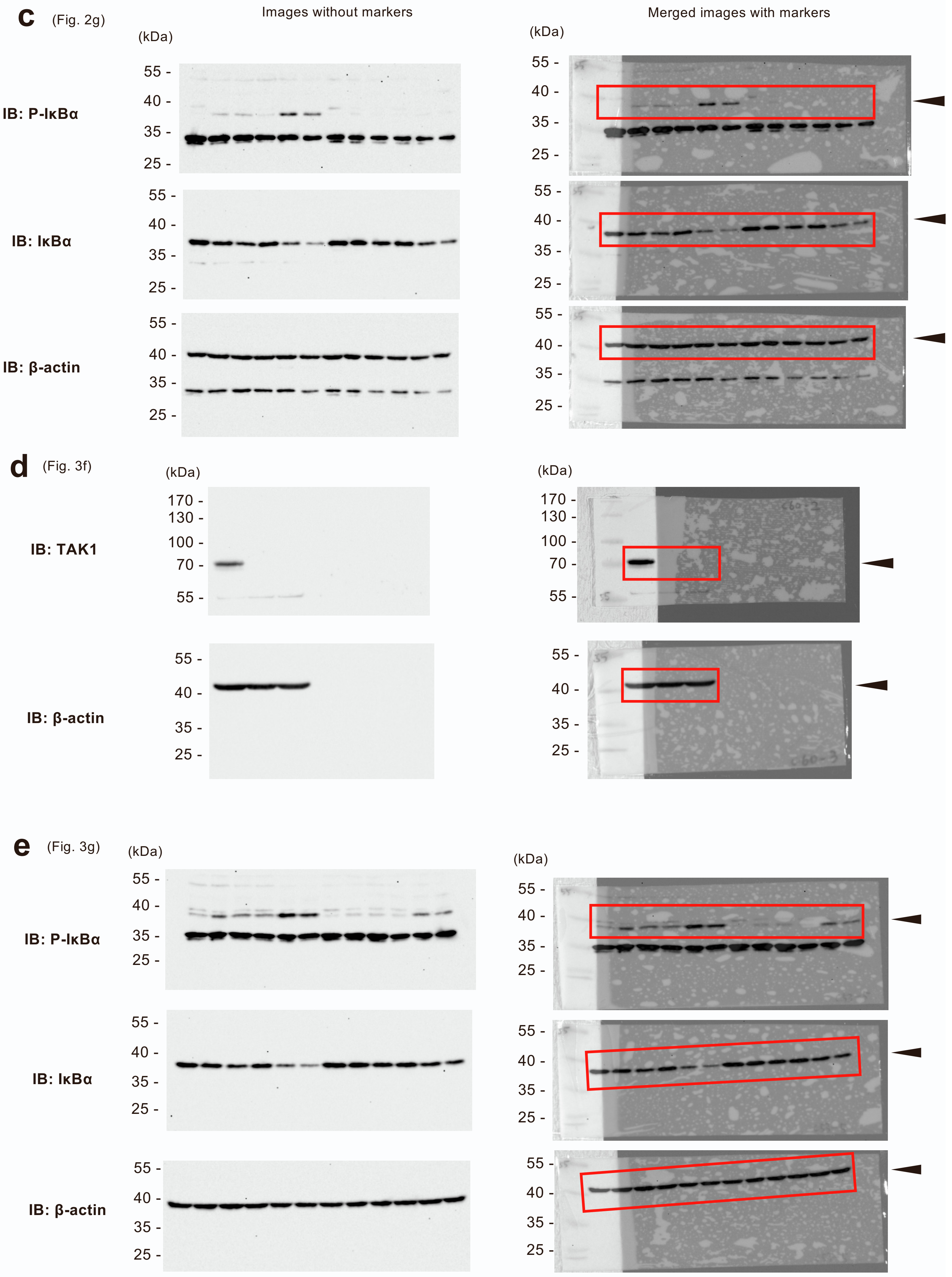

Kojima et al., Fig. S6

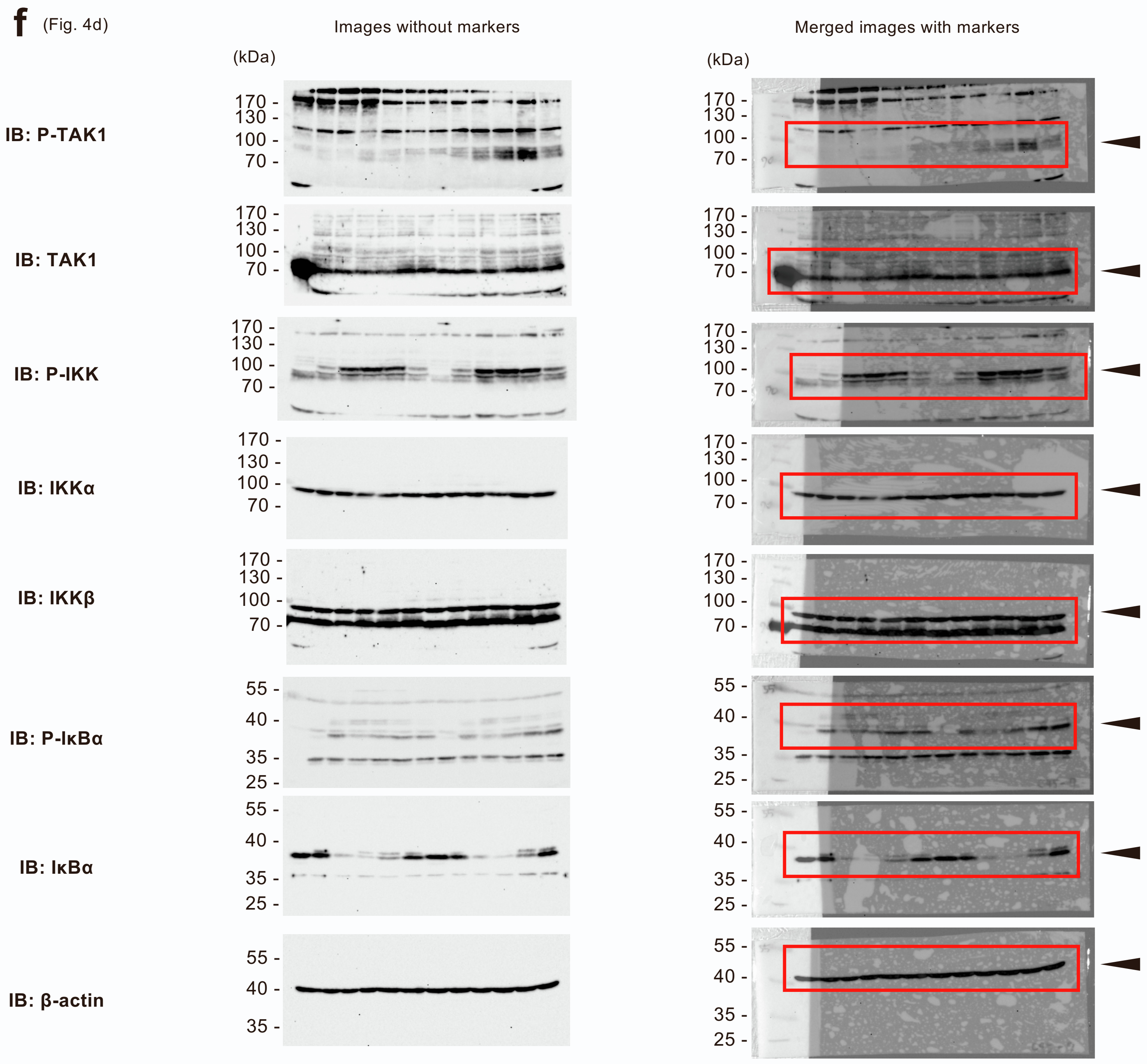

Kojima et al., Fig. S6

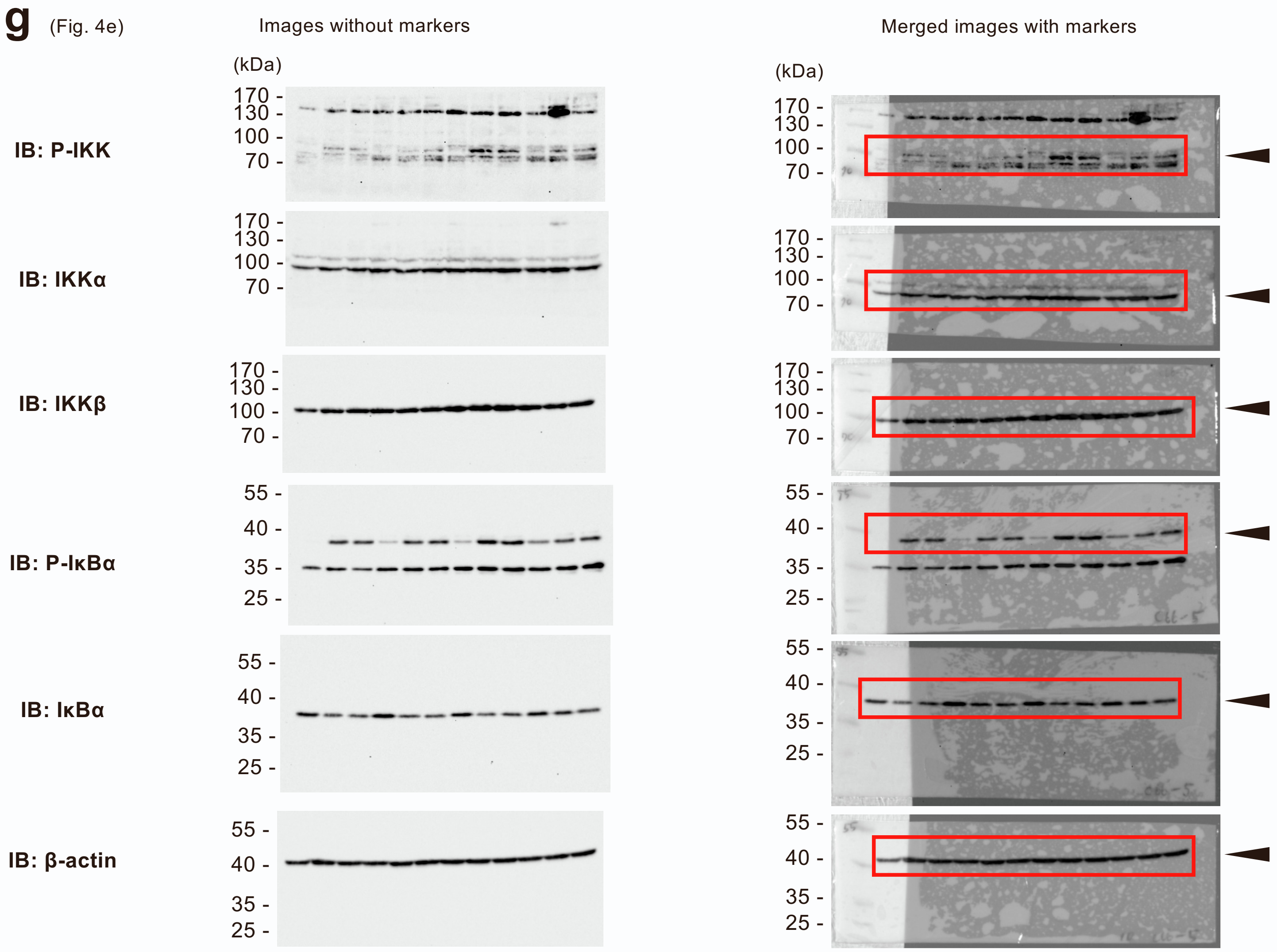

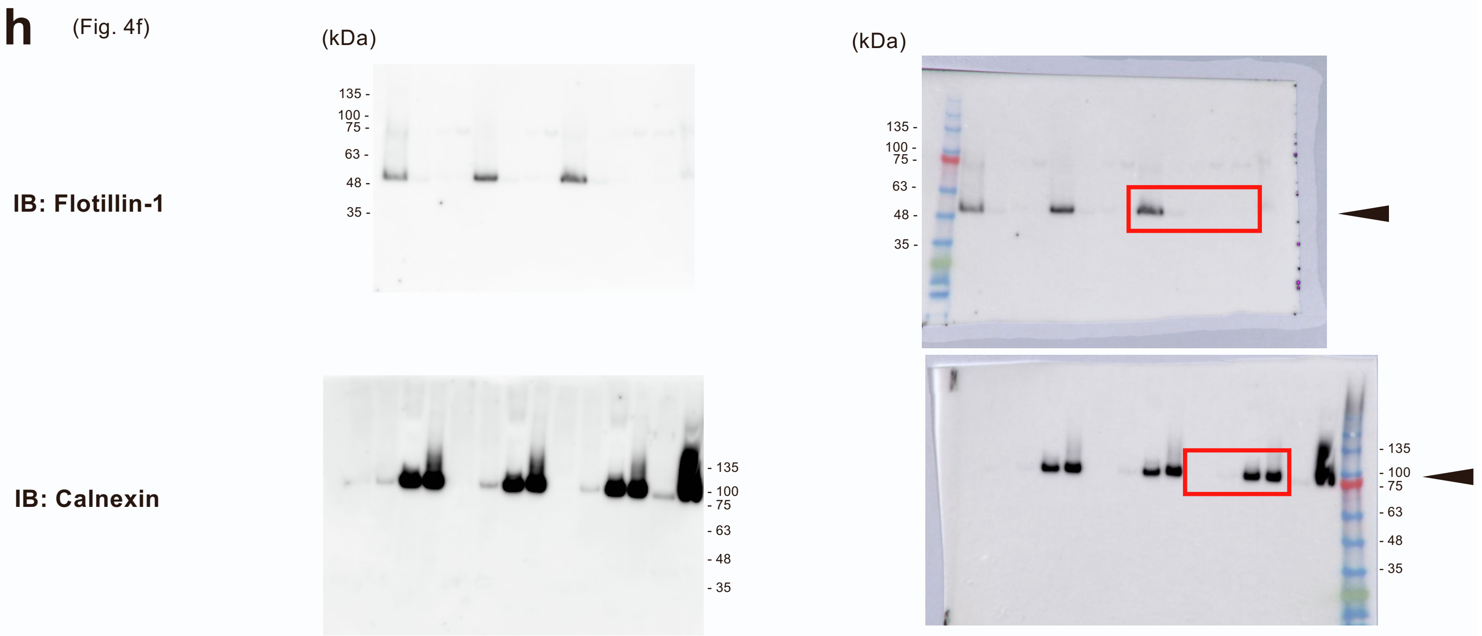

Kojima et al., Fig. S6

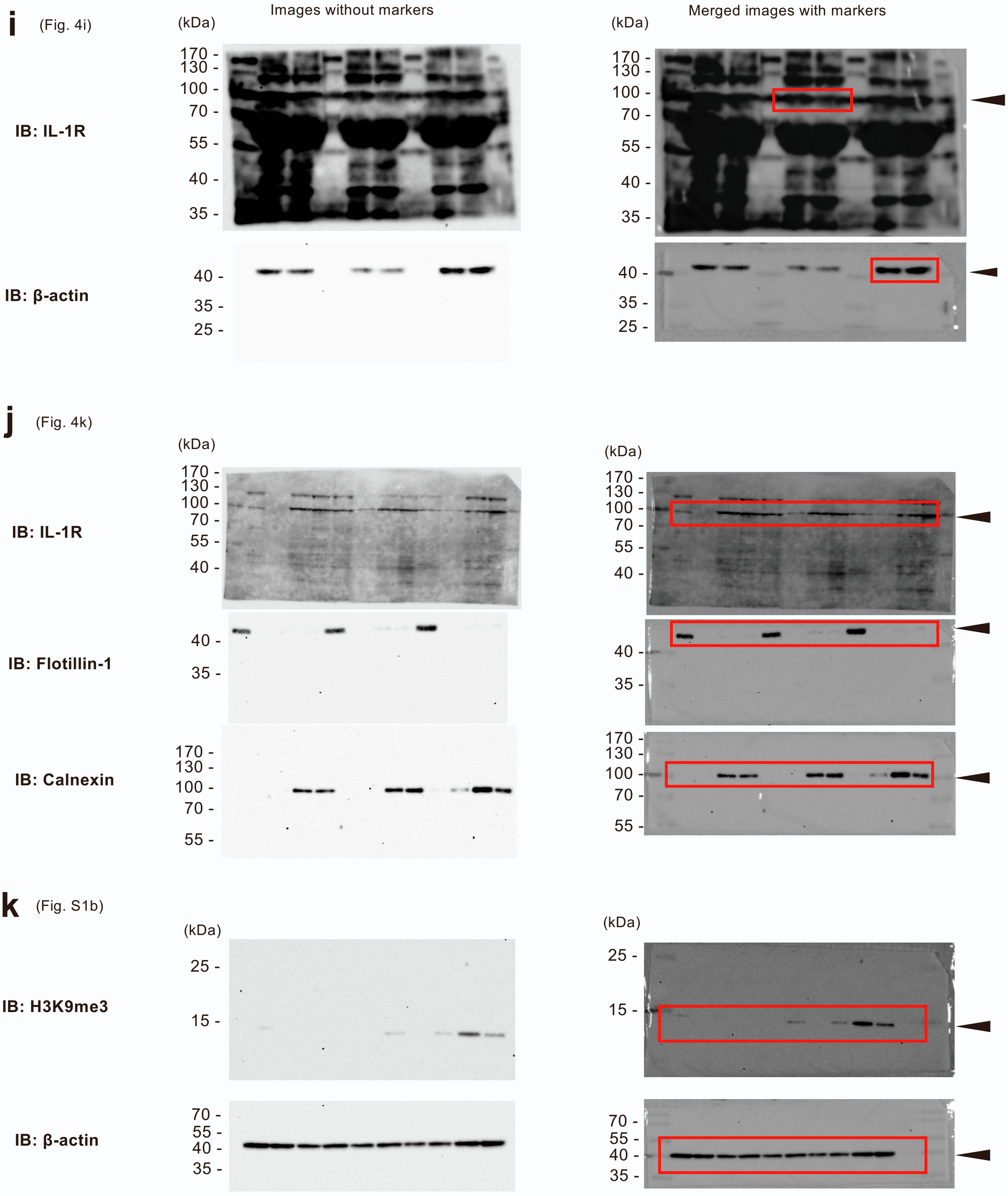

Kojima et al., Fig. S6

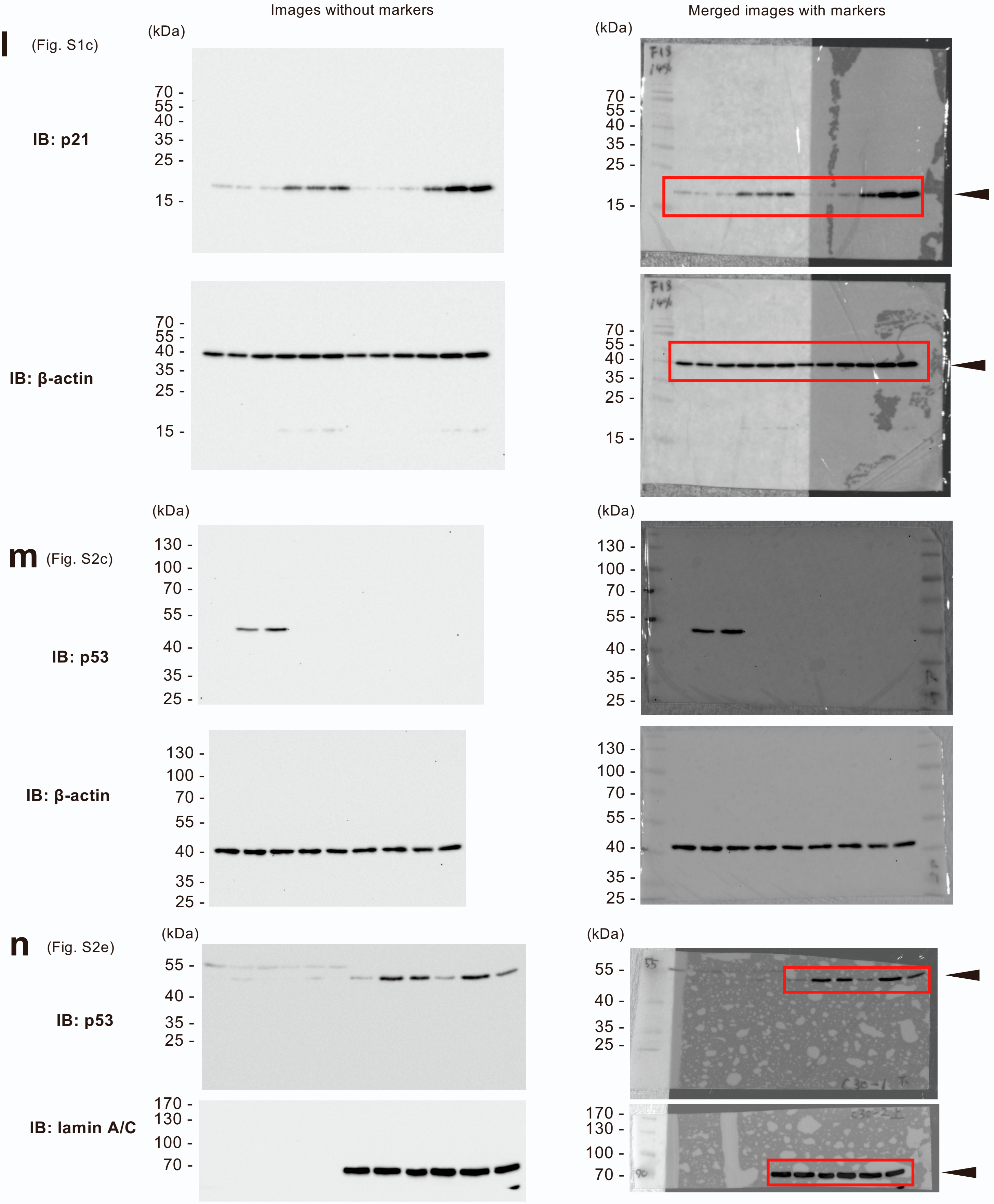

Kojima et al., Fig. S6

o (Fig. S4g)

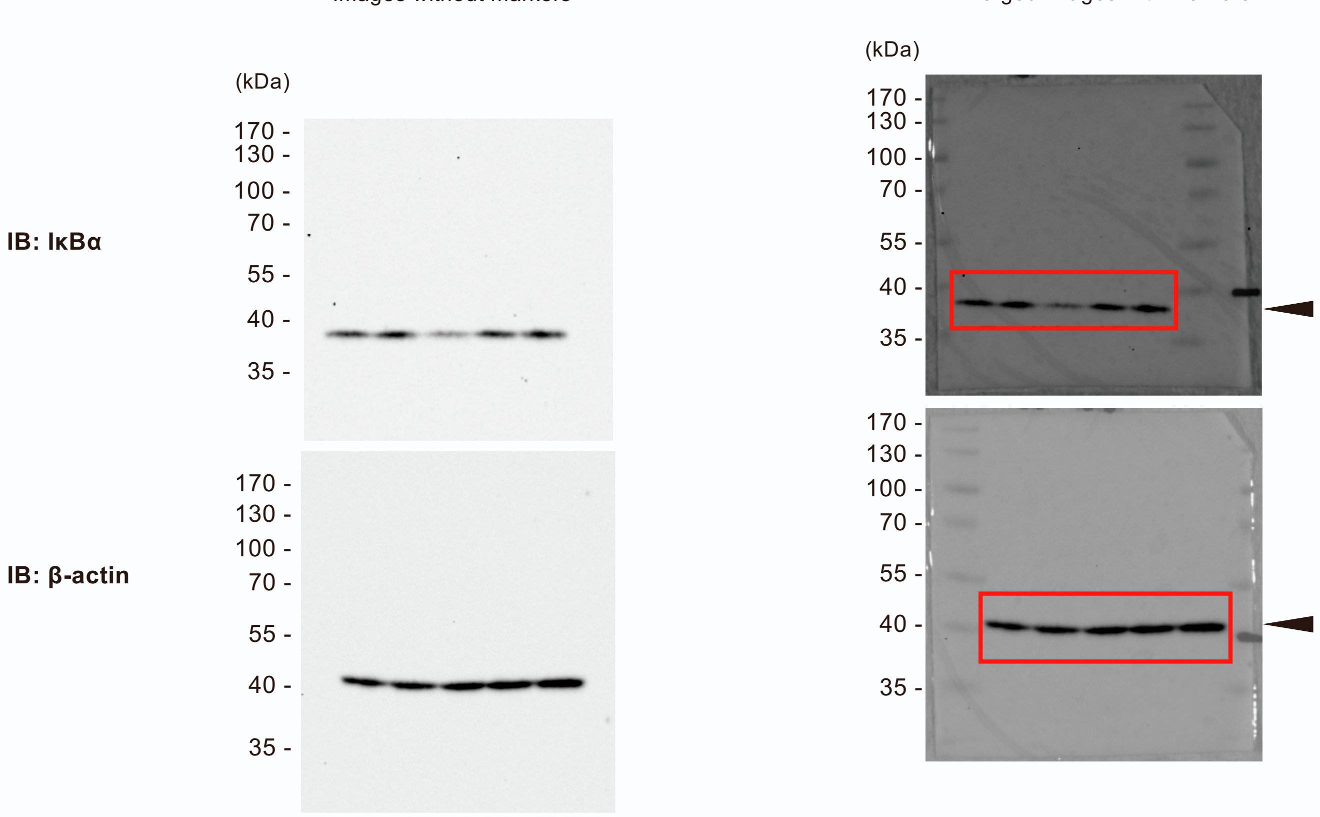

p (Fig. S4h)

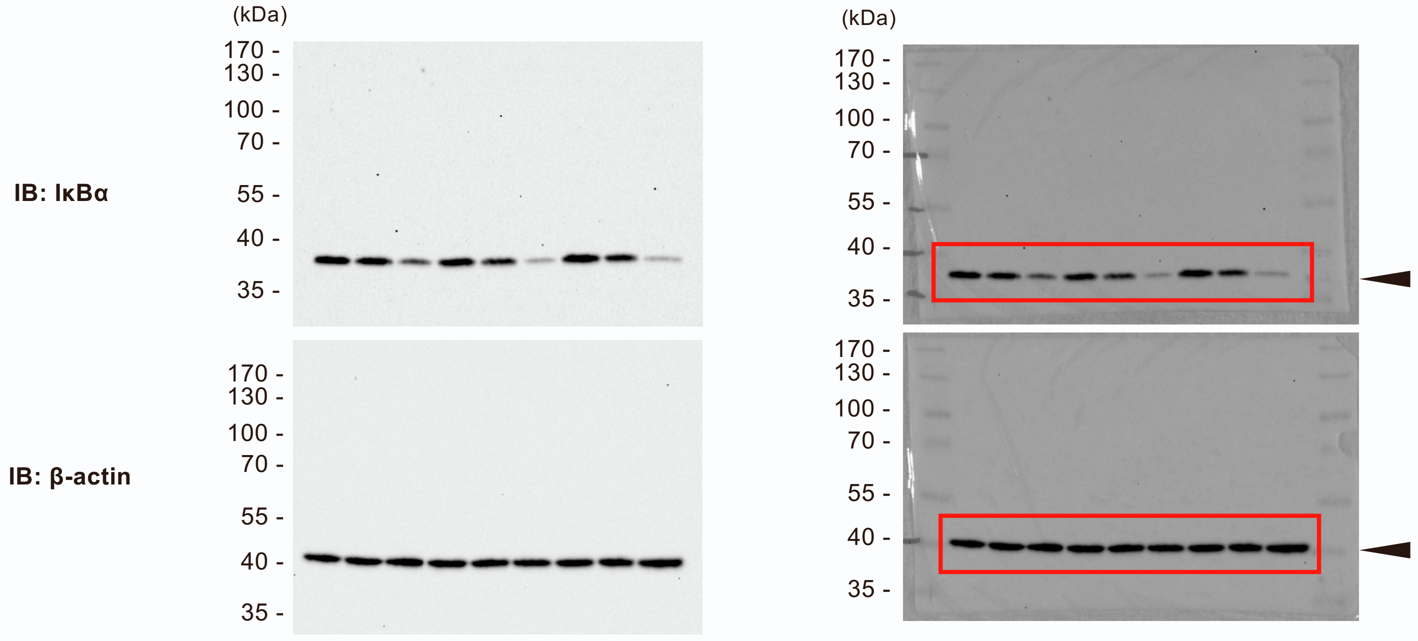

Supplementary Figure 6. Full scans of the immunoblot data

(a-o) Uncropped images of Fig. 2a (a), Fig. 2f (b), Fig. 2g (c), Fig. 3f (d), Fig. 3g (e), Fig.4d (f), Fig. 4e (g), Fig. 4f (h), Fig. 4i (i), Fig. 4k (j), Fig. S1b (k), Fig. S1c (l), Fig. S2c (m), Fig. S2e (n), Fig. S4g (o), Fig. S4h (p)

Table S1: Composition of mouse diets used in this study, related to Figure 5

	NC (#D09100304)		HFD (+EA) (#16010101)		HFD (#09100310N)	
	gm%	kcal%	gm%	kcal%	gm%	kcal%
Protein	22.5	20	22.5	20	22.5	20
Carbohydrate	44.9	40	44.9	40	44.9	40
Fat	19.9	40	19.9	40	19.9	40
Total		100		100		100
kcal/g	3.78		4.49		4.49	
**Ingredient**	**gm**	**kcal**	**gm**	**kcal**	**gm**	**kcal**
Casein	200	800	200	800	200	800
L-Cystein	3	12	3	12	3	12
Corn Starch	350	1400	0	0	0	0
Maltodextrin 10	85	340	100	400	100	400
Fructose	0	0	200	800	200	800
Sucrose	96	384	96	384	96	384
Dextrose	169	676	0	0	0	0
Cellurose	50	0	50	0	50	0
Soybean Oil	25	225	25	225	25	225
Lard	20	180	20	180	20	180
Corn Oil, Partially Hydrogenated	0	0	135	1215	0	0
Palm Oil	0	0	0	0	135	1215
Mineral Mix S10026	13	0	10	0	10	0
DiCalcium Phosphate	10	0	13	0	13	0
Calcium Carbonate	5.5	0	5.5	0	5.5	0
Pattasium Citrate, 1H2O	16.5	0	16.5	0	16.5	0
Vitamin Mix V10001	10	40	10	40	10	40
Choline Bitartrate	2	0	2	0	2	0
Cholesterol	18	0	18	0	18	0
FD&C Yellow Dye #5	0.025	0	0.025	0	0	0
FD&C Red Dye #40	0.025	0	0	0	0	0
FD&C Blue Dye #1	0	0	0.025	0	0	0
**Total**	1073.05	4057	904.05	4056	904	4056

Table S2: Fatty acid composition of mouse diets used in this study, related to Figure 5

	NC (#D09100304)	HFD (+EA) (#16010101)	HFD (#09100310N)
C8, Caprylic	0.000	0.000	0.022
C10, Capric	0.000	0.001	0.022
C12, Lauric	0.000	0.002	0.277
C14, Myristic	0.028	0.028	0.277
C15	0.000	0.002	0.011
C16, Palmitic	0.596	1.508	7.046
C16:1, Palmitoleic, n-9	0.028	0.033	0.055
C17	0.009	0.010	0.022
C18, Stearic	0.289	2.208	0.996
C18:1, Oleic, n-9	1.146	7.232	6.648
C18:1, Elaidic, Trans	0.000	5.063	0.000
C18:2, Linoleic	1.659	2.182	3.319
C18:2, Trans	0.000	0.195	0.000
C18:3, Linolenic	0.196	0.235	0.465
C18:3, Trans	0.000	0.067	0.000
C20, Arachidic	0.009	0.128	0.000
C20:1	0.019	0.097	0.066
C20:2	0.019	0.018	0.044
C20:3, n-6	0.000	0.003	0.022
C20:4, Arachidonic, n-6	0.009	0.007	0.000
C20:4, n-3	0.000	0.000	0.011
C22, Behenic	0.009	0.055	0.000
C22:1, Erucic	0.000	0.000	0.011
C22:5, Docosapentaenoic, n-3	0.000	0.002	0.000
C24, Lignoceric	0.000	0.039	0.000
**Total**	**4.017**	**19.910**	**19.303**
**Saturated**	**0.941**	**3.980**	**8.739**
**Cis-unsaturated**	**3.075**	**9.822**	**10.564**
**Trans-unsaturated**	**0.000**	**5.324**	**0.000**
**kcal (%)**	**10**	**40**	**40**

Table S3: Fatty acid composition of mouse livers after 12 weeks of feeding period, related to Figure 5

	ND	HFD (EA+)	HFD
C16:0	24.052±2.955	19.3375±3.078*	20.538±1.536
C16:1 c9	5.290±0.613	4.960±1.275	4.580±0.941
C18:0	6.176±1.891	2.090+0.737**	2.732±0.873**
C18:1 t9	N.D.	3.303±1.230	N.D.
C18:1 c9	32.164±5.323	47.305±1.579***	44.914±1.300***
C18:1 c11	8.168±1.399	8.665±0.566	4.412±2.591**
C18:2 c9c12	10.386±1.613	6.283±1.906**	14.342±1.521**
C20:4 c5, c8, c11, c14	5.836±1.918	1.800±1.483*	2.600±1.172
C22:6 c4, c7, c10, c13, c16, c19	3.892±1.752	1.340±1.048*	1.602±0.646

Table S4: List of all the oligo sequences used in this study, related to STAR Methods

primers/siRNAs/gRNAs	Sequences (5' to 3')
qRT-PCR primers	
gapdh Fw	TGTGTCCGTCGTGGATCTGA
gapdh Rv	CCTGCTTCACCACCTTCTTGAT
IL-6 Fw	TACCCCCAGGAGAAGATTCC
IL-6 Rv	TTTTCTGCCAGTGCCTCTTT
IL-8 Fw	CAGTTTTGCCAAGGAGTGCTAA
IL-8 Rv	AACTTCTCCACAACCCTCTGC
p21 Fw	GAGGCCGGGATGAGTTGGGAGGAG
p21 Rv	CAGCCGGCGTTTGGAGTGGTAGAA
IL-1R Fw	GGCCAGTTGAGTGACATTGC
IL-1R Rv	AGGTAGACCCTTCCCCAACA
IL-1α Fw	AACCAGTGCTGCTGAAGGA
IL-1α Rv	TTCTTAGTGCCGTGAGTTTCC
col1a1 Fw	ACGTGGAAACCCGAGGTATG
col1a1 Rv	CTTGGGTCCCTCGACTCCTA
IL-1β Fw	GAAATGCCACCTTTTGACAGTG
IL-1β Rv	CTGGATGCTCTCATCAGGACA
cd14 Fw	GCCTTTCTCGGAGCCTATCT
cd14 Rv	TGGCTTCGGATCTGAGAAGT
timp1 Fw	CATGGAAAGCCTCTGTGGATATG
timp1 Rv	AAGCTGCAGGCACTGATGTG
siRNA	
IL-1RI #1	GGUGGAGGAUUCAGGACAU
IL-1RI #2	GCAGCAUAUAUCCAGUUAA
gRNA target sequences and genotyping primers	
p53 target sequence	GATCTGAGCAGCGCTCATGG
p53 Fw	TCCAAATACTCCACACGCAA
p53 Rv	CTACAAGCAGTCACAGCATATG
Raptor target sequence	GAGTCGTAGACGAAGATCGAC
Raptor Fw	TCACCAGCAGCCCATATTCC
Raptor Rv	TTCTGAGCCCTGCTCCATTC
TAK1 target sequence	GTCCCTGTGAATTAGCGCTT
TAK1 Fw	TTCGGGGTGGTGAGAGTGA
TAK1 Rv	TTGTGCCTTTCTTTCGCAGT
